# Mouse Genetic Models of Human Brain Disorders

**DOI:** 10.3389/fgene.2016.00040

**Published:** 2016-03-23

**Authors:** Celeste Leung, Zhengping Jia

**Affiliations:** ^1^The Hospital for Sick Children, Program in Neurosciences and Mental Health, Peter Gilgan Centre for Research and Learning, TorontoON, Canada; ^2^Program in Physiology, University of Toronto, TorontoON, Canada

**Keywords:** genetic models, transgenic mice, gene targeting, knockout, knock-in, inducible, brain disorders, behavior

## Abstract

Over the past three decades, genetic manipulations in mice have been used in neuroscience as a major approach to investigate the *in vivo* function of genes and their alterations. In particular, gene targeting techniques using embryonic stem cells have revolutionized the field of mammalian genetics and have been at the forefront in the generation of numerous mouse models of human brain disorders. In this review, we will first examine childhood developmental disorders such as autism, intellectual disability, Fragile X syndrome, and Williams-Beuren syndrome. We will then explore psychiatric disorders such as schizophrenia and lastly, neurodegenerative disorders including Alzheimer’s disease and Parkinson’s disease. We will outline the creation of these mouse models that range from single gene deletions, subtle point mutations to multi-gene manipulations, and discuss the key behavioral phenotypes of these mice. Ultimately, the analysis of the models outlined in this review will enhance our understanding of the *in vivo* role and underlying mechanisms of disease-related genes in both normal brain function and brain disorders, and provide potential therapeutic targets and strategies to prevent and treat these diseases.

## Autism Spectrum Disorders

Autism spectrum disorders (ASDs) are amongst the most prevalent childhood disorders, affecting 1 in every 68 children within North America (Bolivar, 2014). ASD is a developmental disability that includes a broad spectrum of neurodevelopmental diseases including autism, Asperger’s disorder, Rett syndrome, childhood disintegrative disorder, and pervasive developmental disorder. Amongst these, autism remains the most common ASD. As characterized by DSM-V, children diagnosed with autism have severe social interaction impairments, accompanied by concrete social communication deficits, and stereotyped or repetitive behaviors. Therefore, as one of the leading socioeconomic healthcare problems, there has been ongoing effort to identify genetic biomarkers and to elucidate their mechanisms to treat the triad of impairments associated with this neurodevelopmental disorder. Through genetic screening and post-mortem studies, various candidate genes have been identified to be associated with autism. This section will primarily focus on four well-studied mouse models relevant to autism and the behavioral outcomes relevant to this complex disorder. These models are prime examples of how targeting autism-related genes in mice can be a useful tool to investigate the cellular and molecular processes underlying the behavioral consequences of this complex disorder.

### Neurexins

The neurexin family (NRXN1–3) of presynaptic plasma membrane proteins are *trans*-synaptic cell adhesion molecules that connect functionally pre- and post-synaptic neurons at synapses (Sudhof, 2008; Rabaneda et al., 2014). The extracellular domain of NRXNs interacts with post-synaptic neuroligins (NLGN1–4) across the synaptic cleft and have an essential role in synaptic regulation (Sudhof, 2008; Rabaneda et al., 2014). Each NRXN gene in mammals is composed of differentially distributed long α- and short β-NRXN isoforms. Together, NRXNs and NLGNs are required for proper synapse maturation and *trans-*synaptic neurotransmission (Varoqueaux et al., 2006; Sudhof, 2008). Current gene association studies reveal that hypofunction of NRXN isoforms, specifically rare single gene variations in NRXN1, are linked to autism. Since, 0.5% of all ASD cases appear to harbor NXRXN-1α gene deletions, Etherton et al. (2009) generated NRXN1α knockout (KO) to uncover the behavioral and functional consequences of this gene deletion. NRXN1α KO mice were generated by deleting the promoter and large first exon of the murine α-NRXN genes (Tabuchi and Sudhof, 2002; Missler et al., 2003). These KO mice were found to have impaired excitatory synaptic strength, decreased prepulse inhibition, increased grooming behavior, and enhanced motor learning (Etherton et al., 2009). The authors proposed that NRXN1α is responsible for regulating a proper excitatory to inhibitory balance in synaptic transmission and various behavioral phenotypes related to autism. In addition, recent studies demonstrate that the heterozygous deletion of NRXN1α also resulted in impaired social memory salient with behaviors relevant to autism (Dachtler et al., 2015). In another study, Rabaneda et al. (2014) generated a deletion mutant to target the NRXN1β isoform that lacks the cytoplasmic tail to uncouple NRXN1β *trans-*synaptic function. Transgenic hemagglutinin (HA)- tagged βNRXN1 ΔC were expressed in an inducible manner under the control of a tetracycline-responsive promoter element (TRE). The TRE-HA βNRXN1 ΔC mice were crossed with a CaMKIIα-tTA promoter to restrict the expression of the mutant protein in mature glutamatergic neurons in the forebrain. As expected, the double transgenic CaMKIIα-tTA TRE-HA βNRXN1 ΔC mice expressed the mutant protein in forebrain neurons of the cortex and striatum (Rabaneda et al., 2014). These mice displayed increased self-grooming and deficits in social interactions, consistent with the triad of autism-related symptoms (Rabaneda et al., 2014). However, with the administration of the tetracycline analog, doxycycline (DOX), in the drinking water, the expression of HA- βNRXN1 ΔC was suppressed. The autistic-like phenotypes were reversed following DOX administration thereby suggesting that NRXNs regulate functional and behavioral activity in mature neurons. These results suggest that the presynaptic NRXN1 may cause autistic phenotypes via interacting with its post-synaptic partner NLGNs, altering excitatory synaptic function.

### Contactin-Associated Protein 2

Contactin-associated protein 2 (CNTNAP2), a member of the NRXN super family, encodes a neuronal transmembrane protein (CASPR2) involved in the formation of tight axonal connections critical for the establishment and maintenance of neuronal circuits (Rodenas-Cuadrado et al., 2014; Gdalyahu et al., 2015). CASPR2 is localized in juxtaparanodes of myelinated axons and mediates interactions between neurons and glia during development (Poliak et al., 1999). *In vitro* studies have shown that RNAi-mediated knock-down of CASPR2 impairs the development and maturation of dendritic spines resulting in decreased synaptic transmission (Anderson et al., 2012). Moreover, the loss of function of CASPR2 led to impaired synaptic connectivity and neural network assembly. Alterations in the CNTNAP2 gene are strongly linked to language deficits in complex neurological disorders including autism, specifically in patients with a single nucleotide polymorphism in intron 2 of the CNTNAP2 gene (rs7794735; Arking et al., 2008). In mice, ablation of CNTNAP2 resulted in fewer GABAergic interneurons, neuronal migration abnormalities, and reduced cortical neuronal synchrony (Penagarikano et al., 2011). In addition, it was found that the intermodal localization of Kv1 channels at the juxtaparanodal region depends on the presence of CNTNAP2 (Gordon et al., 2014). Behaviorally, CASPR2 KO mice exhibited core autism-like symptoms including lower levels of sociability and decreased ultrasonic vocalizations with increased levels of grooming, hyperactivity, and deficits in reversibility on the Morris water maze test. In a recent study, CNTNAP2 KO mice were found to have reduced spine density due to increased spine elimination and impaired stabilization of new spines (Gdalyahu et al., 2015). Therefore, these results together suggest the importance of CNTNAP2 in neuronal migration and formation of cortical neural networks that may underlie the behavioral responses. In particular, the decreased GABAergic interneuron activity may imply that neural asynchrony or an excitatory and inhibitory imbalance may be a pathophysiological mechanism to explain how information processing is impaired in autism.

### Neuroligins

Neuroligins (NLGN1–3, 4X, 4Y) are a family of post-synaptic cell adhesion molecules that are ligands of their *trans-*synaptic partner NRXN (Ichtchenko et al., 1995; Autism Genome Project et al., 2007; Kim et al., 2008). Human genetic association studies have linked changes in the NLGN genes with autism (Sudhof, 2008; Glessner et al., 2009). Therefore, mouse models that target each of the different NLGN isoforms have been generated to better understand the *in vivo* function of these genes and how their mutations contribute to autism-like behaviors. KO mice lacking individual NLGNs were created through the deletion of exon sequences spanning the translational start site and 380 bp of the 5′ coding sequence of the respective genes via homologous recombination in ES cells (Varoqueaux et al., 2006). The deletion of the NLGN1 gene, whose expression is localized at excitatory synapses, resulted in decreased NRXN1 levels, reduced excitatory transmission, and selective deficits in social memory, spatial learning and memory tests, and increases in grooming (Blundell et al., 2010). However, these authors did find a 30% increase in the expression of NLGN3, suggesting a functional compensation between members of the NLGN family (Blundell et al., 2010). Moreover, these KO mice exhibited deficits in hippocampal long term potential (LTP), the most extensively studied form of long-lasting synaptic plasticity widely regarded as the cellular mechanism for learning and memory, which was accompanied by reduced NMDA/AMPA ratios (Blundell et al., 2010; Jedlicka et al., 2013). Together, these results suggest that NLGN1 is important in cognitive and repetitive behaviors characteristic of autism although the relationship between LTP, NMDA receptor-mediated synaptic transmission, and the observed behavioral deficits remains unclear. Other studies have examined the role of NLGN1 in a transgenic mouse model under a ubiquitous Thy1-promoter to drive neuron-specific overexpression throughout the brain (Hines et al., 2008; Dahlhaus et al., 2010). These transgenic mice exhibited deficits in LTP and impairments in memory acquisition on the Morris water maze test, which further corroborates the importance of NLGN1 in synaptic plasticity and cognitive processes (Hines et al., 2008). Recent studies have shown that the conditional KO (CKO) of NLGN2 in the adult medial prefrontal cortex results in the gradual loss of inhibitory synapses (Liang et al., 2015). The authors deleted NLGN2 locally via bilateral stereotactic injection of AAVs expressing GFP tagged Cre-recombinanse. The mice exhibited impairments in anxiety-like characteristics, fear memory, social interaction behaviors, and alterations in inhibitory synaptic properties (Durieux et al., 2015; Liang et al., 2015). The overexpression of NLGN2, which is exclusively expressed at inhibitory synapses, resulted in enlarged inhibitory synaptic contact size in the frontal cortex and an overall reduction in the ratio between excitatory and inhibitory synaptic activity (Hines et al., 2008). Furthermore, these animals displayed hyperactivity, increased stereotypic jumping, elevated anxiety-like behavior, and impaired social interactions but normal sociability (Hines et al., 2008). Together, these results confirm that NLGN2 plays an essential role at the inhibitory synapse and suggest that it may also contribute to ASD-like behaviors. Perhaps the best studied mouse model based on the NLGN genes that is relevant to autism are the NLGN3 knock-in (KI) mice harboring the R451C missense mutation (i.e., Arg451 is substituted with Cys451) because this mutation was shown to be linked to autistic patients. The NLGN3 R451C KI mice had a 90% decrease in NLGN3 protein levels in the forebrain based on immunoblotting analysis (Tabuchi et al., 2007). In addition, there was a slight decrease in the level of the NLGN1 isoform. The NLGN3 R451C KI mice exhibited increases in inhibitory synaptic transmission and deficits in social interactions but enhanced spatial learning and memory. Thus, the R451C substitution of NLGN3 acts as a gain-of-function mutation consistent with patients with ASD (Jamain et al., 2008). Other studies have demonstrated impaired tonic endocannabinoid signaling that is responsible for the altered inhibitory transmission (Foldy et al., 2013). Consistent with NLGN3 R451C KI, NLGN3KO mice (Varoqueaux et al., 2006) display behavioral phenotypes relevant to autism; including reduced ultrasonic vocalizations, a lack of social novelty preference, and a decrease in total brain volume (Radyushkin et al., 2009). Recent studies indicate that in both NLGN3 R451C KI and KO mice, there appear to be changes in synaptic mechanisms within the striatum that facilitate repetitive behaviors associated with this disease (Rothwell et al., 2014). NLGN4 KO mice were generated with a gene trap insertion 340 bp downstream of the first exon of NLGN4 (first coding sequence) by BayGenomics. Since a small segment of the essential esterase domain remains, NLGN4 is unable to bind to NRXNs and therefore non-functional (Jamain et al., 2008). These KO mice had selective reductions in reciprocal social interaction and less ultrasonic vocalizations in males upon the presentation of anestrus female (Ey et al., 2012). Recent studies have also reported pronounced perturbations of γ-oscillatory network activity that is implicated in cognition (Hammer et al., 2015). To examine if there is a functional redundancy among the NLGN isoforms, Varoqueaux et al. (2006) generated NLGN1–3 triple KO (TKO) mice lacking their respective signal sequences and extracellular esterase-like domain to abolish NLGN gene expression completely. They chose to focus on NLGN1–3 due to the high expression levels in the brains of newborn mice. It is interesting to note that while NLGN3 is present at both excitatory and inhibitory synapses, NLGN1 and NLGN2 appear to be exclusively expressed at excitatory and inhibitory synapses respectively. These homozygous TKO mice had apparent irregular breathing movements resulting in respiratory failure and death shortly after birth (Varoqueaux et al., 2006). Furthermore, impaired glutamatergic and GABAergic synaptic transmission led to reduced excitatory drive and low spontaneous synaptic activity (Varoqueaux et al., 2006). Therefore, these results suggest that NLGN1–3 are critical for maintaining the excitatory and inhibitory balance for proper synaptic activity. In summary, the results from the mouse models based on altered NLGN genes are consistent with the notion that changes and mutations in the NLGN genes contribute to autistic phenotypes likely through altered synaptic properties, including abnormal excitatory/inhibitory balance, synaptic plasticity, and endocannabinoid signaling.

### SH3 and Multiple Ankyrin Repeat Domains (Shanks)

The Shank family (Shank1–3) of genes code for post-synaptic scaffolding proteins with multiple protein-protein interaction domains (Naisbitt et al., 1999). These domains include a Src homology 3 domain (SH3), ankyrin repeats domain (ANK), post-synaptic density 95 PSD-95/disks large/zone occludens-1 domain, a proline-rich region, a homer-binding region, a cortactin-binding region, and a sterile alpha motif (Naisbitt et al., 1999). The Shank proteins are localized to the post-synaptic density and are important in coordinating the assembly of signaling complexes at glutamatergic synapses (Bockers et al., 2001; Durand et al., 2007). Mutations in Shank3 have been strongly implicated in ASD compared to the other two Shank family genes, and therefore, various lines of Shank3-mutant mice have been generated to investigate the functional role of each of the domains and its contribution to the neuropathology of ASD. One of the first studies used the Cre/loxP system to disrupt the exons coding for the ANK of Shank3 thereby allowing regional-specific targeting of the gene (Bozdagi et al., 2010). Specifically, the loxP sites were inserted to flank the ANK and the floxed region was then excised by crossing it to CMV-Cre transgenic mice. The Shank3-deficient mice displayed reduced basal glutamatergic synaptic transmission and LTP along the Schaffer collateral pathway and impaired reciprocal social interactions including reduced social behavior reflected through conspecific sniffing and vocalizations (Bozdagi et al., 2010). Recent studies demonstrate that the homozygous deletion of the ANK result in a significant reduction in NMDA/AMPA ratio at the excitatory synapses onto striatal medium spiny neurons and impairments in LTP (Jaramillo et al., 2015). The haploinsufficiency of Shank3 or the loss of one functional copy of the Shank3 gene results in chromosome 22q13 deletion syndrome or Phelan-McDermid syndrome and autism (Phelan et al., 2001; Uchino and Waga, 2013). Studies have genetically targeted Shank3α, the longest Shank3 isoform that is responsible for encoding the ANK without affecting Shank3β and Shank3γ isoforms (Peca et al., 2011). Shank3α KO mice were found to have modest ASD-like behavioral outcomes. Peca et al. (2011) also created a second mouse line that targeted another isoform, Shank3β, through the deletion of the exons coding for the fragment containing the PSD-95/disks large/zonula occludens-1 or PDZ domain. It is important to note that in this study, the Shank3α-and β- isoforms were completely eliminated and the Shank3γ- isoform was only significantly reduced (Peca et al., 2011). These Shank3αβ KO mice were found to have normal gross anatomy but displayed anxiety-like behaviors and compulsive repetitive grooming behavior that resulted in self-inflicted skin lesions (Peca et al., 2011). However, individually, on tests of social interaction, Shank3β KO mice were less sociable, while Shank3α KO mice displayed mild impairments in social novelty recognition (Peca et al., 2011). In a recent study, Speed et al. (2015) knocked-in a single guanine nucleotide into exon 21 of the Shank3 gene, which mimics an autism associated insertion mutation and results in a frameshift causing a premature Shank3 protein. These mice displayed spatial learning deficits, impaired motor coordination, and altered novelty and sensory processing salient with the cardinal features of autism (Speed et al., 2015). Therefore the results from the Shank3 mice are consistent with the idea that deletions/mutations at the Shank3 gene are contributing factors for ASD through regulating synaptic function at excitatory synapses.

## Intellectual Disability

Intellectual disability is one of the most devastating neuro-developmental disorders in children and young adults. ID involves impairments in general mental abilities that affect conceptual, social, and practical domains of everyday tasks. Children with ID often have IQ scores approximately two standard deviations below the population (Mandel and Chelly, 2004). This pervasive disorder manifests itself beginning at early development and comorbid with other neurological conditions including ASD, depression, and attention-deficit/hyperactivity disorder. The prevalence of ID has been gradually increasing in the last decade and said to affect 3% of the population (Chelly and Mandel, 2001). Due to the severity and the social/economic effect of the disease, numerous attempts have been made to better understand the development and behavior in these children. However, the complexity of other symptoms including comorbid features renders it difficult to interpret the genetic and environmental factors involved in the pathophysiology of ID. ID is classified into two primary categories based on the clinical presentation, syndromic and non-syndromic ID, which allows different candidate genes to be tested for genotype and phenotype comparisons. Syndromic ID includes all patients present with one or multiple clinical features or comorbidities, whereas non-syndromic ID describes patients with ID as the sole clinical feature. In this section, we will discuss selected mouse models for both syndromic and non-syndromic ID and illustrate how genetically altered mice are used to study the etiology of ID.

### p21-Activated Kinases

In the mammalian central nervous system, PAKs (p21-activated kinases) are a family of serine/threonine protein kinases that are activated by Rho-family small GTPases, such as RhoA, Rac, and Cdc42 (Field and Manser, 2012). The PAK family is divided into group I and II kinases based on the presence of an autoinhibitory domain (AID). Since group II PAKs lack an AID, binding by upstream Rho-family GTPases does not always lead to consistent activation of the kinase (Manser et al., 1995; Baskaran et al., 2012). Group I PAKs are involved in multiple cellular processes particularly actin regulation, cell proliferation, apoptosis, and gene expression and alterations in PAK1–3 are associated with tumor formation, inflammation, and cognitive dysfunction (Kumar et al., 2006; Zhao et al., 2006). Recent studies have demonstrated that 25–35% of ID have genetic associations with nonsense or missense mutations on the X chromosome, some of which result from loss of function in the PAK3 gene (Kelly and Chernoff, 2012). A number of mouse models have thus been generated based on the PAK genes to investigate the *in vivo* role of these genes and how their mutations contribute to ID (Allen et al., 1998; Boda et al., 2004; Dubos et al., 2012). Hayashi et al. (2004) generated a transgenic mouse model to inhibit the catalytic activity of group 1 PAKs in the postnatal forebrain. The dominant-negative PAK (dn-PAK) transgene, which consisted of an α-CaMKII promoter, amino acids 74–146 encoding the AID of PAK3, and SV40 intron/polyA, was used to create the mouse model (Hayashi et al., 2004). The dnPAK transgenic mice displayed decreased spine density and increased proportion of large synapses in cortical pyramidal neurons. Surprisingly, these mice showed enhanced AMPAR-and NMDAR-mediated synaptic transmission and LTP (Hayashi et al., 2004). Behaviorally, the dnPAK mice exhibited specific impairments in the consolidation and retention of hippocampal-dependent memory in a fear conditioning paradigm. These data suggest that PAKs in general play a role in synaptic structure and plasticity and cognitive processes. PAK1 and PAK3 single KO mice were previously created by replacing a part of the coding and adjacent upstream/downstream intronic sequence by a pgk-neomycin resistant cassette to completely eliminate the entire kinase domain of PAK1 and 3 respectively (Meng et al., 2005; Asrar et al., 2009). PAK1 KO mice revealed normal brain anatomy but selective deficits in LTP at hippocampal CA1 synapses with changes in levels of actin binding protein, cofilin (Asrar et al., 2009). PAK3 KO mice resulted in no deficits in either cofilin activity or the actin cytoskeleton with mild impairments in late-phase LTP and learned taste aversion (Meng et al., 2005). In a recent study, Huang et al. (2011) investigated the role of PAKs by analyzing double knockout (DKO) mice lacking both PAK1 and PAK3 to eliminate functional redundancy. The DKO mice were generated by crossing previously characterized single PAK1 and PAK3 KO mice (Meng et al., 2005; Asrar et al., 2009). The DKO mice exhibited impaired postnatal brain growth, deficits in dendritic arborization and spine morphology, with alterations in synaptic transmission and plasticity (Huang et al., 2011). Furthermore, the DKO mice displayed a host of behavioral deficits consistent with ID-like symptoms including hyperactivity, increased anxiety, and learning and memory deficits. The alterations in spines were rescued by blocking cofilin activity suggesting cofilin-dependent actin regulation may mediate the effect of PAK1 and PAK3. Actin, the major cytoskeletal component in dendritic spines, is known to regulate a host of synaptic properties particularly post-synaptic receptor trafficking and spine morphology (Meng et al., 2004). Since the genetic deletions of either PAK1 or PAK3 in mice have only produced modest effects, PAK1 and PAK3 appear to have at least some overlapping functions in synaptic and cognitive regulation (Meng et al., 2005; Asrar et al., 2009; Huang et al., 2011). Further, analyses of these single and double KO mice will be needed to elucidate the *in vivo* roles and detailed mechanisms of PAKs. In addition, mouse models with specific PAK gene mutations relevant to ID will be required to translate animal studies to human conditions. Ultimately, these studies will help to reveal cellular and molecular mechanisms involved in the pathogenesis of non-syndromic x-linked ID.

### Fragile X Syndrome

Fragile X syndrome is the most commonly inherited form of ID and the most common cause of autism (O’Donnell and Warren, 2002; Hayashi et al., 2007; Pietropaolo and Subashi, 2014). FXS is a genetic diagnosis affecting 1 in 1250 males and 2500 females (Crawford et al., 2001). More specifically, FXS is a multi-organ disease with widespread effects leading to ID, macroorchidism in males, and premature ovarian insufficiency in females (Brennan et al., 2006; Ascano et al., 2012). FXS patients exhibit an increased incidence of seizures, anxiety, depression, and subtle motor impairments (Berry-Kravis et al., 2007; Basuta et al., 2011; Chonchaiya et al., 2012). This neurodevelopmental disorder is caused by the loss of the Fragile X mental retardation protein (FMRP) encoded by the Fragile X mental retardation 1 (FMR1) gene (Verkerk et al., 1991). Clinical studies reveal that patients with FXS have increased expansion and hypermethylation of trinucleotide (CGG) repeats within the promoter of FMR1 (Fu et al., 1991; Kremer et al., 1991a,b). FMRP is predominantly expressed within the cytoplasm of cells and have been implicated in translational processes including RNA interference and RNA subcellular localizations by facilitating nucleo-cytoplasmic shuttling (Devys et al., 1993; Eberhard and Grummt, 1996; Fridell et al., 1996). Human studies have revealed that FXS patients have abnormal development and morphological changes such as increased abundance of long, thin, and immature dendritic spines (Hinton et al., 1991; Irwin et al., 2001). The molecular mechanisms governing the spine and behavioral anomalies observed in FXS are not clear but analyses of FRMP mouse models based on the FMR1 gene have provided significant progress, which will be discussed below.

#### Fragile X Mental Retardation Protein

In order to examine the effects of the FMRP, the Dutch-Belgian Fragile-X Consortium engineered a mouse model in which the wild-type murine FMR1 gene was knocked out through introducing a neomycin cassette (Bakker et al., 1994; No authors, 1994). The FMR1KO mice displayed similar phenotypic characteristics seen in patients with FXS; including hyperactivity, increased anxiety and depressive-like states, cognitive deficits, and macroorchidism in male mice (Bregman et al., 1988; Bakker et al., 1994; No authors, 1994; Kooy et al., 1996; Yuhas et al., 2011). In a recent study, FMR1KO mice further showed reduced social discrimination between a familiar and novel mouse (Sorensen et al., 2015). However, FMR1KO mice lack FMRP expression in the entire animal. Thus to specifically investigate the spatial and temporal expression patterns of FMRP, the design of more novel and versatile KO models were needed. Mientjes et al. (2006) used the Cre/loxP system to disrupt the exons coding for FMR1 to achieve Purkinje cell-specific expression. The floxed region was then excised by crossing it to pcp-2/L7-Cre expressing mice to generate conditional FMR1KO (CKO) mice (Mientjes et al., 2006). These FMR1CKO mice revealed deficits in classical cerebellar-mediated delay eye-blink conditioning (Koekkoek et al., 2005). Amiri et al. (2014) used Nse-Cre (neuron-specific enolase promoter-driven Cre transgene) mice to drive loxP-specific deletion of FMR1 in subsets of neurons in the mouse cortex and hippocampus at 4 weeks of age. The FMR1CKO mice showed elongated spines and cell physiological abnormalities at the parallel fiber synapses that form on these spines (Amiri et al., 2014). Due to the similar sequence homology and tissue distribution, FXR2P, a homolog of the FMRP, has been suggested to compensate in part for the function of FMRP which may explain the mild phenotypes observed in FMR1KO mice (Kirkpatrick et al., 2001; Bontekoe et al., 2002; Mientjes et al., 2006). FXR2KO mice were created through inserting a neomycin resistant gene to study the function of FXR2P. Similar to FMR1KO mice, FXR2KO mice exhibited hyperactivity accompanied by mild disruptions on the rotarod test (Bontekoe et al., 2002; Spencer et al., 2006). However, FXR2KO mice exhibited less contextual conditioned fear, reduced prepulse inhibition, and learning deficits on the Morris water maze test, suggesting that FXR2 and FMR1 may play different roles in FXS phenotypes (Bontekoe et al., 2002). To examine if FXR2 and FMR1 have overlapping functions, other studies have created the FMR1/FXR2 double KO (DKO) mice through crossing individual FMR1KO and FXR2KO mice (Bontekoe et al., 2002). These DKO mice demonstrate increased anxiety, hyperactivity, reduced prepulse inhibition, and deficits in spatial and associative learning and memory suggesting a functional relationship between FXR2P and FMRP (Spencer et al., 2006). Together, these different methods of targeting FMRP and FXR proteins outline how these interactions can contribute to FXS phenotypes. However, further analyses are needed to elucidate the detailed cellular and molecular mechanisms involved in the pathogenesis of FXS.

### Williams- Beuren Syndrome

Williams- Beuren syndrome (WS) is a neurodevelopmental disorder caused by the deletion of a 1.5-million-bp-segment on chromosome 7 (7q11.23; Pober, 2010). This microdeletion is caused by misalignment of chromosome 7, a region spanning 28 genes flanked by highly homologous clusters of genes during meiosis that results in unequal homologous recombination and consequently deletion (Pober, 2010). To date, WS is a rare disorder that affects approximately 1 in 7500 births (Stromme et al., 2002; Sanders et al., 2011). Patients with WS exhibit a broad range of cognitive and systemic deficits. These include congenital heart disease, an ID profile with an unusual pattern of abilities (IQ of approximately 64 but are concrete in vocabulary and verbal short-term memory), and weaknesses in visuospatial construction and long-term memory (Mervis and John, 2010; Mervis and Velleman, 2011). Genetic association studies have implicated a variety of genes to contribute to the cognitive and behavioral profile of WS. However, only a few of these targets that reside in the commonly deleted hemizygous region have exhibited consistent cognitive and behavioral deficits of WS when translated to a mouse model. These include LIMK1 (Frangiskakis et al., 1996), CLIP2 (Hoogenraad et al., 2002), GTF2I (Merla et al., 2010), and GTF2IRD1 (Merla et al., 2010). Although, several genetic models have been produced and characterized for these genes, we will focus on LIMK1 KO mice that partially recapitulate the behavioral and cognitive profile of WS.

#### Lim Domain Containing Kinase

Lim domain containing kinase 1 (LIMK1) is a serine/threonine protein kinase and a potent regulator of actin dynamics via phosphorylating and thus inactivating the actin-depolymerization factor (ADF)/cofilin (Arber et al., 1998; Yang et al., 1998; Sumi et al., 1999). *In vitro* studies have indicated that the Rho family small GTPases, Rac1 and Cdc42, bind and activate PAKs and ROCK protein kinases, which phosphorylate and activate LIMK1 (DesMarais et al., 2005; Jia et al., 2009; Huang et al., 2011). The activated LIMK1 can directly phosphorylate and inactivate cofilin, which promotes actin polymerization (DesMarais et al., 2005; Jia et al., 2009; Huang et al., 2011). Human genetic studies reveal that WS patients with smallest microdeletions containing the LIMK1 gene (Tassabehji et al., 1996) show some key WS phenotypes (i.e., visuospatial deficit), suggesting that LIMK1 may be a key factor in WS pathology. To test this hypothesis, LIMK1KO mice were generated replacing a part of the second LIM and PDZ domains with a pgk-neomycin resistant cassette, to completely eliminate the kinase domain (Meng et al., 2002). These mice had normal expression of other proteins including LIMK2, PAKs, ROCK2, and cofilin, but reduced cofilin phosphorylation and altered actin networks and spine morphology. Behaviorally, the LIMK1KO mice displayed deficits in locomotion, fear responses, and visuospatial cognition (Meng et al., 2002). Recent studies have also identified that LIMK1KO were particularly impaired in long-lasting LTP (specifically late-phase LTP) and the formation of long-term memory (Todorovski et al., 2015). In addition, LIMK1KO mice were found to have a reduction in active (phosphorylated) CREB and increasing CREB activity rescued the long-term memory deficits in these mice suggesting that impaired CREB signaling may be responsible for late-LTP and long-term memory deficits. Taken together, these studies indicate that the deletion of LIMK1 is sufficient to cause synaptic dysfunction and impaired long-term memory that may be relevant to WS. Therefore, LIMK1 is likely a key factor in WS pathology. Furthermore, these studies also suggest that LIMK1 regulates synaptic plasticity and behavior via two distinct mechanisms; cofilin-mediated actin reorganization and CREB-mediated gene expression. Ultimately, these studies may provide insight into the neural circuitry and molecular targets for treating and preventing WS.

## Schizophrenia

Schizophrenia is a chronic neuropsychiatric disorder characterized by positive and negative symptoms, including delusions, hallucinations, disorganized speech, catatonic behavior, diminished emotional expression, and impaired cognition (Abazyan and Pletnikov, 2014). Given the severity and heterogeneity of these core symptoms, schizophrenia is deemed to be one of the most debilitating diseases by the World Health Organization, affecting approximately 1% of the entire population. The onset of schizophrenia is in post-adolescence (16–25 years), but the course of the illness and presentation of core symptoms can persist into adulthood (40–60 years). Although genetic and environmental factors contribute to the etiology of schizophrenia, no specific biomarkers have been identified for this multifactorial disorder. In a recent study Ayalew et al. (2012) used a translational convergent functional genomics approach to identify and prioritize genes involved in schizophrenia. Through, gene-level integration of genome-wide association data combined with gene expression studies in humans and animal models, they identified top candidate genes including: DISC1, NRG1, RELN, and many others (Ayalew et al., 2012). Their findings shed light on novel promising targets for future pharmacological treatments for patients with schizophrenia. In this section, we will discuss two well-characterized genetic mouse models of schizophrenia that have recreated structural and behavioral dysfunctions associated with the disease. In the end of this section, we will briefly discuss alternate models that have been used to examine the effects of dopamine and glutamate neurotransmission in schizophrenia.

### Disrupted-in-Schizophrenia 1

Disrupted-in-Schizophrenia 1 (DISC1) is a scaffolding protein with numerous binding partners that facilitate the formation of protein complexes (Morris et al., 2003; Camargo et al., 2007). In humans, DISC1 is 854 amino acids long consisting of a N-terminal globular head domain and a long helical C-terminal coiled tail domain (Chubb et al., 2008; Soares et al., 2011). DISC1 is expressed systemically in the heart, liver, kidney, and thymus, but highly enriched in the cerebral cortex, hippocampus, hypothalamus, amygdala, cerebellum, and olfactory bulbs in the brain (Ma et al., 2002; Austin et al., 2004; Brandon et al., 2009). The DISC1 gene was first identified as a risk factor for mental disorders including schizophrenia, schizoaffective disorder, recurrent major depression, and adolescent conduct and emotional disorder, in a Scottish pedigree that displayed a translocation between chromosomes 1 and 11 (q42;q14.3; St Clair et al., 1990). Since then, other studies have confirmed that the translocation t(1;11) disrupts the DISC1 gene in several populations, that are also susceptible to similar psychiatric illnesses (Sachs et al., 2005; Chubb et al., 2008). The first DISC1 mouse model was engineered based on a spontaneous 25-bp deletion in exon 6 of the DISC1 gene in a 129S6 strain that abolished the production of the full-length DISC1 protein (Koike et al., 2006). The DISC1-deficient mice exhibited cognitive dysfunction specifically in spatial working memory (Koike et al., 2006). In recent studies on the same DISC1-deficient mice, Kvajo et al. (2011), found widespread and cumulative cytoarchitectural alterations in the dentate gyrus during neonatal and adult neurogenesis. In particular, they noticed errors in axonal targeting and impaired dendritic growth that are accompanied by changes in short-term plasticity at mossy fiber/CA3 synapses (Kvajo et al., 2011). Furthermore, Juan et al. (2014) revealed subtle working memory impairments were mediated by decreased neuronal excitability and morphological alterations in layer II/III pyramidal neurons in the medial prefrontal cortex in the same DISC1-deficient mice. These studies suggest that the DISC1 gene from the 129S6/SvEv strain is important for neuronal connectivity, short-term synaptic dynamics and working memory. Other models have been established based on mutations in the mouse DISC1 gene via *N*-nitroso-*N*-ethylurea (ENU) mutagenesis, which induces point mutations at a high locus-specific rate (Coghill et al., 2002). Clapcote et al. (2007), identified and analyzed two independently derived ENU-induced missense mutations in the 955-bp exon 2 of mouse DISC1, known as Q31L and L100P. They chose to focus on exon 2 due to the fact that it is the longest, present in all known splice isoforms, and encodes most of the head domain of the DISC1 protein (Millar et al., 2005; Clapcote et al., 2007). The Q31L mice displayed depressive-like behavior or learned helplessness in the forced swim test (FST), reduced sociability, and low sucrose consumption indicative of anhedonia (Clapcote et al., 2007). The L100P mice exhibited schizophrenic-like behavior with profound deficits in prepulse inhibition and latent inhibition, common methods to quantify information-processing deficits. These L100P mice also had greater horizontal locomotion and decreased working memory performance on the T-maze task (Clapcote et al., 2007). The deficits observed in L100P mice could be reversed by pharmacological or genetic inactivation of glycogen synthase kinase 3 (GSK-3α and GSK-3β; Clapcote et al., 2007). Similarly, the Q31L deficits in prepulse inhibition and FST could be rescued following a GSK-3 blocker, TDZD-8, suggesting a role for GSK-3 in mediating the effects of DISC1 (Lipina et al., 2012). Recent studies have shown that the Q31L mice have fewer proliferating cells, whereas the L100P mice have differences in the generation, placement, and maturation of newly generated neurons in the adult hippocampus (Chandran et al., 2014). Despite these differences, both models share structural and functional brain abnormalities, particularly reductions in overall brain volume due to tissue contraction in the neocortex, thalamus, and cerebellum. Thus, both the Q31L and L100P mice exhibit impairments that are consistent with the positive, negative, and cognitive symptoms of schizophrenia. In recent studies, Lee et al. (2013) discovered altered interneuron density and location in the L100P mice due to selective impairments in calbindin- and parvalbumin-expressing interneurons in the cortex and hippocampus, and consequently decreased GAD67/PV co-localization in the neocortex, suggesting a role of inhibitory transmission in schizophrenia. Lastly, there have also been other transgenic models that result in the expression of mutant DISC1 in a spatial-temporal manner. Hikida et al. (2007) have exogenously expressed dominant-negative truncated DISC1 (DN-DISC1) under the regulation of an α-CaMKII promoter that is restricted in the olfactory bulb, frontal cortex, hippocampus, and basal ganglia. In this particular model, DN-DISC1 did not affect the expression level of the endogenous DISC1 gene (Hikida et al., 2007). Similar to patients with schizophrenia, the DN-DISC1 mice displayed enlarged lateral ventricles contributing to asymmetrical brain anatomy, interneuron deficits resulting in cortical synchrony, and impairments in prepulse inhibition, FST, and spontaneous activity. Therefore, analysis of the DISC1 mouse models has contributed significantly to and will continue to provide new insights to our understanding of the *in vivo* function of the DISC1 gene and how its alterations result in schizophrenia.

### Neuregulin-1

The family of neuregulins, also known as NDF, heregulin, GCF, and ARIA (NRG1–4), are epidermal growth factors (EGFs) that activate ErbB receptor tyrosine kinases, and play diverse roles in normal developmental processes, plasticity, and oncogenesis (Burden and Yarden, 1997; Harrison and Law, 2006; Britsch, 2007). Neuregulin-1 (NRG1), the best characterized amongst the four neuregulins, was discovered in 1992 and shown to have diverse functions in the heart, breast, and nervous system (Holmes et al., 1992; Harrison and Law, 2006). In the brain, NRG1 modulates neuronal migration, glial development and differentiation, and synaptic transmission and plasticity (Harrison and Law, 2006). In 2002, NRG1 was identified as a candidate gene for schizophrenia in a genome-wide linkage scan conducted by Stefansson et al. (2002). In this study, the NRG1 gene was mapped to the chromosome 8p locus (8p11-p21), a region linked to schizophrenia. Therefore, numerous mouse models have been generated to examine the functional consequences of targeting NRG1 and its receptor, ErbB. To target the EGF-like domain, exon 6 of the NRG1 gene was fused to β-galactosidase (NRG1-LacZ), which resulted in the partial deletion of the EGF-like domain of all three isoforms of NRG1 (Meyer and Birchmeier, 1995). Also, a second vector was used that targeted exons 7–9, which encode parts of the EGF domain (carboxy-terminus) of NGR1, was replaced by a neomycin resistance gene (Meyer and Birchmeier, 1995). The matings between heterozygous mice carrying either mutation produced no homozygous mutant offspring, indicating that the complete deletion of NRG1 and its receptor, ErbB, are lethal during embryogenesis due to heart malformations and defects in Schwann cells and cranial glia development (Meyer and Birchmeier, 1995). Therefore, NRG1 and its ErbB receptor are indispensable during development. In subsequent studies, the NRG1 heterozygous mice were therefore used and found to exhibit significant behavioral alterations such as hyperactivity in the open field test, improved rotorod performance, and enhanced spontaneous alternation ability in T or Y-maze tests (Gerlai et al., 2000; Stefansson et al., 2002; O’Tuathaigh et al., 2007). Other studies have shown that hyperactivity and increased locomotion in NRG1 heterozygous mice can be reversed with clozapine (an antipsychotic to treat schizophrenia) and environmental enrichment (Karl et al., 2007; Duffy et al., 2008). Therefore, the targeted deletion of NRG1 provides novel insights into the role of NRG1 and supports that it may contribute to schizophrenia. Several studies have also examined the effects of increased NRG1 expression in transgenic mouse models. Kato et al. (2010) constructed a transgenic vector that contained the promoter of a house keeping gene, elongation factor 1α (EF-1α) and GFP-tagged NRG1 cDNA that drives ubiquitous expression in the whole brain. They confirmed through Western blot and real-time quantitative reverse transcription that the overexpression of NRG1 was 4.3-fold higher than wild-type littermates (Kato et al., 2010). Behaviorally, the transgenic mice had elevated locomotor activity, decreased context-dependent fear conditioning, and impaired social interaction in an isolation-induced resident-intruder test (Kato et al., 2010). Moreover, the mice had an increased expression of GABAergic paravalbumin and myelination markers in the frontal cortex, suggesting a role for NRG1 in inhibitory transmission. Therefore, the mouse models based on NRG1 presented in this section help to identify the behavioral and mechanistic processes that may be relevant to the pathophysiology of schizophrenia.

Although, we have discussed two well-characterized genetic models used for studying the pathophysiological mechanisms of schizophrenia, other studies exist that attribute the pathological mechanisms of schizophrenia to dopamine (DA) and glutamatergic neurotransmission dysfunction. The DA hypothesis of schizophrenia proposes that hyperactive DA transmission in the striatum contributes to positive symptoms of schizophrenia while cognitive functions and negative symptoms may result from hypoactive DA neurotransmission in the prefrontal cortex (Carlsson and Lindqvist, 1963; Davis et al., 1991; Howes and Kapur, 2009). Animal models have thus been created to evaluate the roles of D2 receptors in neurotransmission via inducible overexpression in the striatum alone (Kellendonk et al., 2006). The D2 transgenic mice exhibited selective cognitive impairments in working memory tasks that may be relevant to schizophrenia (Kellendonk et al., 2006). In the second hypothesis, glutamate receptors specifically NMDA receptor hypofunction have been associated with the negative symptoms and cognitive dysfunction similar to schizophrenia (Javitt and Zukin, 1991; Coyle, 2006; Javitt, 2007). Studies have found that hypomorphic NR1 mice with approximately 4% expression of NR1 (via inserting a neomycin resistance gene into intron 20) exhibit complex phenotypes including hyperactivity and decreased social interactions (Mohn et al., 1999; Duncan et al., 2002). Therefore, analysis of these models will be useful to determine the role and underlying mechanisms of DA and glutamate transmission in the pathogenic processes of schizophrenia.

## Alzheimer’s Disease

Alzheimer’s disease is a debilitating age-associated neuro-degenerative disorder, affecting more than 35 million people worldwide (Hall and Roberson, 2012). This neurocognitive disorder results in a decline of intellectual function usually beginning with impaired language usage or communication and ultimately, interferes with one’s ability to function independently (Janus and Borchelt, 2014). Therefore, as one of the most devastating neurodegenerative disorders, potential biomarkers have been developed via brain imaging such as MRI and PET scans, genetic, and biochemical studies. AD has a well-defined neuropathological profile including the presence of extracellular amyloid plaques, accretion of intracellular neurofibrillary tangles (NFTs), neuronal damage, and death in selected brain regions (Arnold et al., 1991; Vigo-Pelfrey et al., 1993; Braak and Braak, 1994; Petersen et al., 1999; Naslund et al., 2000). Genetic linkage and positional cloning strategies have identified various genes associated with AD. These include: amyloid precursor protein, presenilin 1 and 2, α-2 macroglobulin, low-density lipoprotein receptor-related protein and others (Goate et al., 1991; Levy-Lahad et al., 1995; Sherrington et al., 1995; Sperling et al., 2011). In this section, we will discuss three genetic models targeting amyloid-β-precursor protein, presenilin 1 and 2, and tau that are involved in producing the AD phenotype.

### Amyloid-β-Precursor Protein

Amyloid- β-precursor protein (APP) is a type I (single-pass) transmembrane protein with a large amino-terminal extracellular domain (O’Brien and Wong, 2011; Hall and Roberson, 2012). Gene mutations in APP alter the production and deposition of the amyloid-beta peptide (Aβ), which hasten the production of extracellular amyloid plaques (Goate et al., 1991). The Aβ peptides (36–43 amino acids long) are generated from APP by β- and γ-secretase (Lewis et al., 2001). Alternative splicing of the APP transcript generates eight isoforms, but only one (695 amino acid form) is predominantly expressed in the CNS (Bayer et al., 1999). The first genetic model to target APP used the PDGF-β promoter to drive expression of APP717 cDNA gene encoding a variant mutation where valine at residue 717 is substituted by phenylalanine (V717F) that leads to overexpressing 40 copies of the transgene (Games et al., 1995). This missense mutation in APP is tightly linked to autosomal dominant forms of AD (Games et al., 1995). These and other transgenic mice [i.e., the ones using similar Thy1-APP (V717F)] displayed a ten-fold elevation of APP protein and resulted in deficits in reference memory on the radial arm maze and spatial memory on the Morris water maze test, and an accumulation of amyloid in the brain between 6 and 7 months (Games et al., 1995; Dodart et al., 1999; Moechars et al., 1999). Therefore, overexpression of this mutant APP is sufficient for the formation of amyloid plaques in mice. However, other anatomical anomalies and their mechanisms were not investigated including dystrophic neurites, the formation of NFTs, and reduced synaptic density and function. Perhaps one of the best characterized animal studies of AD is the Tg2576 transgenic mouse model, which overexpresses a mutant APP containing Swedish FAD (K670N/M67L) double mutations at the two β-secretase cleavage sites responsible for Aβ production (Hsiao, 1998). Compared to the previous model, the Tg2576 mice have moderate levels of APP overexpression (5–6 fold of the endogenous level; Hsiao, 1998). The mice had a slow accumulation of amyloid in the neocortex and hippocampus around 12 months (Sturchler-Pierrat et al., 1997; Kawarabayashi et al., 2001) and impaired spatial reference and contextual memory (Comery et al., 2005). Others have engineered transgenic mice harboring the Swedish and London FAD mutations, revealing that these double mutants have a sevenfold overexpression of APP with typical Aβ plaques at the age of 6 months and 14–25% neuronal loss between 14 and 18 months (Calhoun et al., 1998). Behaviorally, these mice showed a broad range of abnormalities that included changes in limb and stereotypic movements, deficits in learning and memory, and electrophysiological impairments in basal synaptic transmission (Roder et al., 2003; Comery et al., 2005). More recent studies have used a modified version of APP where the Aβ peptide-coding domain of the mouse cDNA was humanized (Borchelt et al., 1996). In these mice, APP was expressed three-fold higher than the endogenous APP and were found to develop amyloid plaques at old ages (24–26 months) accompanied by various deficits in cognitive performance (Borchelt et al., 1996; Savonenko et al., 2003). Recent studies have shown that these mice with the overexpression of human APP demonstrate reductions in synapse-associated proteins including PSD95, AMPA and NMDA receptor subunits, and neuronal maker, MAP2, indicating signs of neurodegeneration in the hippocampus (Simon et al., 2009). Therefore, studies of mutant APP transgenic mouse models have indicated that abnormal expression of APP plays a critical role in AD pathology.

### Presenilin

More than 150 familial mutations in presenilin 1 and presenilin 2 (PS1 and PS2) have been identified in AD (De Strooper, 2007). PSs encode the catalytic subunit of γ-secretase that cleaves APP to form Aβ of varying lengths (De Strooper, 2007). AD-associated PS mutations have been shown to increase the two most common isoforms of Aβ, Aβ42 and Aβ40, both of which are known to be responsible for the rate of amyloid deposition (Borchelt et al., 1996; Scheuner et al., 1996). PSs are also known to be involved in cell adhesion, calcium homeostasis, transport, trafficking/localization, and apoptosis (Hall and Roberson, 2012). To target PS1, several transgenic mice expressing different forms of the human mutated PS1 genes were created, to explore whether mutations in PSs cause cells to secrete a greater amount of amyloidogenic Aβ peptides (Elder et al., 2010). The transgenic mice expressing mutant human PS1 [lines Tg(M146L)1, Tg(M146L)76, Tg(L286V)198] showed significant overproduction of Aβ42 and no cognitive deficits in the Morris water maze test at 6 and 9 months of age (Janus et al., 2000). Only mutant PS1 co-expression with a mutant APP (Tg2576) showed early amyloid plaque deposition, neuron loss in the cerebral cortex, and cognitive dysfunction (Arendash et al., 2001; Lok et al., 2013). Taken together, these studies suggest that the full phenotypic expression of mutant PS1 alleles may require the co-expression of other AD-associated genes. PS1 conditional knockout (CKO) mice were generated by crossing floxed PS1 mice with transgenic mice expressing Cre recombinase under a αCaMKII promoter so that PS1 was selectively inactivated in excitatory neurons in the postnatal forebrain (Yu et al., 2001). The CKO mice showed normal basal synaptic transmission and plasticity, but exhibited mild deficits in spatial learning and memory. Other studies have found that the conditional inactivation of PS1 in APP transgenic mice prevented the accumulation of Aβ peptides, the formation of amyloid plaques, and inflammatory responses but failed to ameliorate the contextual memory and synaptic deficits associated with APP transgenic mice (Saura et al., 2005). Conditional PS1 and PS2 double knockout mice (PS CDKO) revealed cortical shrinkage, hippocampal atrophy, and elevated levels of tau hyperphosphorylation that was accompanied by behavioral deficits in hippocampal-dependent learning and memory (Feng et al., 2004; Saura et al., 2005). Therefore, these results define essential roles of PSs in synaptic plasticity, learning and memory, and neuronal survival in the adult cerebral cortex.

### Tau

The genes discussed above focused on recapitulating the Aβ pathology in the formation of extracellular plaques in AD. However, AD also involves the development of intracellular NFTs mainly comprised of hyperphosphorylated forms of the microtubule-associated protein, tau (Lee et al., 2001). Studies have revealed over 30 mutations in the microtubule-associated protein, tau, in AD patients; ranging from missense, deletion, and silent mutations in the coding region, and intronic mutations, influencing the alternative splicing of tau or its protein level (Clark et al., 1998; Hutton et al., 1998; Goedert, 2005). The first tau KO mice were generated by replacing the signals for the start of translation of tau protein with a pgk-neomycin resistant cassette so that only short fragments incapable of binding to microtubule were produced (Harada et al., 1994). In later studies, these tau KO mice exhibited motor deficits and muscle weakness (Ikegami et al., 2000). Dawson et al. (2001) have also generated tau KO mice, on a BL/6/129sv mixed strain, and showed similar locomotor deficits in balance beam tests, but in this latter model, tau KO mice expressed significant cognitive deficits at 12 months. Furthermore, the authors were able to show that primary hippocampal neurons from the tau-deficient mice had delayed maturation in their axonal and neuritic extensions (Dawson et al., 2001). These results suggest that the genetic background of the tau-deficient mice may result in discrepancies on the deleterious effects of axonogenesis. Studies have shown that the ablation of tau ameliorates AD-related synaptic, network, and behavioral abnormalities in transgenic mice expressing human APP (Morris et al., 2015). Transgenic mice expressing human tau containing the P301L mutation (JNPL3) linked to frontotemporal dementia and Parkinsonism have been produced under the mouse prion promoter (Lewis et al., 2000). These mice were found to show moderate early behavioral deficits in escape extension during tail elevation and spontaneous back paw clenching (Lewis et al., 2000). In addition, these mice developed a progressive motor abnormality due to the loss of lower motor neurons (Lewis et al., 2000). In a later study, when the JNPL3 mice were crossed with the Tg2576 mice, there was an increase in the formation of filamentous tau inclusions and plaques in the forebrain regions compared to JNPL3 mice (Lewis et al., 2001). Behaviorally, these double transgenic mice developed motor disturbances associated with spinal cord and neuromuscular pathology. More recent findings have proposed that post-translational modifications such as acetylation and ubiquitination of endogenous tau, that is not bound to microtubules and is free to interact with diverse molecules in different neuronal compartments, mediates Aβ-induced neuronal impairments (Morris et al., 2015). To study the neuropathological correlates of AD, the interaction between Aβ and tau and their effect on synaptic function was examined in a triple transgenic mouse model (Oddo et al., 2003). Rather than crossing three independent lines of mice, Oddo et al. (2003) co-microinjected human tau (P301L) and Swedish FAD APP mutation (both under the control of a Thy1.2 neuron-specific regulatory element) into single-cell embryos from homozygous PS1(M146V) KI mice. They successfully found five of the six founder lines harbor all three mutations and in two lines the transgenes appeared to cointegrate at the same genetic locus (Oddo et al., 2003). The tight linkage coupled to the PS1 knockin site allowed the mice to breed as a “single” transgenic line (Oddo et al., 2003). The mice exhibited synaptic deficits (LTP) that were manifested in an age-dependent manner, followed by plaques at 3 months of age and NFTs by 12 months in the cerebral cortex, hippocampus, and amygdala (Oddo et al., 2003). These results are consistent with the amyloid cascade hypothesis. Ultimately, this triple Tg-AD mouse along with other AD models described above will provide valuable tools to evaluate the interaction and pathogenic mechanisms of various AD genes. Other models such as those based on Apolipoprotein E (ApoE) gene are also important to assess the involvement of glial cells in AD pathology, but will not be discussed here.

## Parkinson’s Disease

Age-related neurodegenerative diseases also include PD, which is becoming increasingly prevalent in our society. It is estimated that 10 million people worldwide are affected by PD (Hirtz et al., 2007). According to DSM-V, PD is a progressive neurodegenerative disorder clinically characterized by the cardinal symptoms of resting tremor, bradykinesia or the slowness of movement, cogwheel rigidity, and postural instability (Jankovic, 2008). Non-motor symptoms such as the presence of Lewy bodies and neurites that consist of aggregated forms of the 140 amino acid protein α-synuclein were also found in post-mortem brain studies and therefore considered to be one of the histological hallmarks of PD (Fearnley and Lees, 1991; Spillantini et al., 1998; Braak et al., 2003). As the second most common neurodegenerative disorder following AD, PD also attracts a great deal of clinical and basic research. PD is characterized by the loss of dopaminergic cells in the substantia nigra that project to the striatum leading to nigrostriatal degeneration and motor symptoms (Forno, 1996), but the mechanisms underlying this cell loss remain unclear. Genetic models based on gene mutations linked to the monogenic form of familial PD have been essential tools for the mechanistic and treatment studies. In this section, we will discuss three different mouse PD models that have been used in the attempt to evaluate possible neuroprotective treatments and biomarkers for early diagnosis and disease-state identification. It is important to note that none of the current models have been able to precisely replicate all motor and non-motor symptoms, as well as Lewy body formation and nigrostriatal degeneration in an age-related progressive manner.

### α-Synuclein

Alterations in α-synuclein (encoded by the SNCA gene) cause a rare form of autosomal dominant PD. Mutations in α-synuclein is known to be the most common risk factor for idiopathic and familial PD (Pankratz et al., 2009; Satake et al., 2009). PD-related point mutations (such as A30P and A53T), rare triplications, and duplications in the SNCA gene have been identified in human familial PD (Polymeropoulos et al., 1997; Ahn et al., 2008; Gasser, 2009). Therefore, various wild-type or mutant α -synuclein transgenic mice have been developed to examine the consequences of overexpression of the SNCA gene under different promoters. Specifically, mice overexpressing human wild-type α-synuclein under the platelet-derived growth factor-β (PDGF-β) promoter (Line D) exhibited intraneuronal α-synuclein aggregates in the neocortex, hippocampus, and olfactory bulb (Masliah et al., 2000). Furthermore, these mice had lower striatal levels of tyrosine hydroxylase (required for the synthesis of dopamine) enzymatic activity associated with the degeneration of nigrostriatal dopaminergic neurons (Masliah et al., 2000). Behaviorally, the mice displayed rotarod and spatial memory deficits. Thus, intraneuronal accumulation of α-synuclein appears to be required for dopaminergic and behavioral deficits to become detectable (Masliah et al., 2000; Masliah and Hansen, 2012). Another model was generated that overexpressed mutant α-synuclein under the Thy1 promoter, which had a more widespread expression of α-synuclein in neurons of the thalamus, basal ganglia, substantia nigra, and brainstem (Rockenstein et al., 2002). In this model, the human A53T mutation in α-synuclein was introduced into the transgene (van der Putten et al., 2000; Rockenstein et al., 2002). These A53T transgenic mice showed late-onset pathological changes that include axonal degeneration in long white matter tracts of the spinal cord, the breakdown of myelin sheaths, and the degeneration of neuromuscular junctions with a loss of integrity of the presynaptic neurofilament network (van der Putten et al., 2000; Rieker et al., 2011). In an another study, Plaas et al. (2008), generated a mouse model with a subtle point mutation in α-synuclein that replaced alanine with proline at position 30 (A30P). The A30P mice exhibited an age-dependent decline in motor ability on the beam walk test and in mean stride length from all paws (Plaas et al., 2008). Furthermore, the SNCA A30P mice had deficiencies in vmat2, a vesicular transporter that regulates dopamine availability, which indicates that the motor defects may be due to a decreased content of dopamine in the striatum. Double transgenic mice harboring the A30P/A53T mutations displayed progressive decline with age in locomotor activity and stereological counts of tyrosine hydroxylase-positive neurons (Thiruchelvam et al., 2004). In addition to overexpression studies, others have also engineered α-synuclein KO mice. The KO mice were generated by using a floxed neomycin cassette to disrupt the SNCA gene and subsequently crossed with C57BL/6NTacGt(ROSA)26Sortm16(cre)Arte mice (Chandra et al., 2004). These KO mice had normal ultrasynaptic structure and survival rates but displayed decreased striatal dopamine levels (20%), elevated lactate concentrations, and reduced rearing (Chandra et al., 2004; Drolet et al., 2004). These findings suggest that α-synuclein may play a role in the maintenance of presynaptic vesicular function. Chandra et al. (2005) show that transgenic expression of α-synuclein ameliorates the lethality and rapidly progressive neurodegeneration caused by the deletion of cysteine-string protein-α (CSPα), a synaptic vesicle protein essential for neuronal survival, in mice. The expression of transgenic α-synuclein improved SNARE complex assembly, which suggests that α-synuclein acts in conjunction with CSPα to play an important role in protecting presynaptic terminals against neurodegeneration. Triple αβγ-synuclein KO mice that lack all murine synucleins (generated through breeding previously characterized αβ-synuclein KO mice with γ-synuclein KO mice) present age-dependent alterations in synaptic structure and transmission, neuronal dysfunction, diminished survival, and late-onset phenotypes including retinal dysfunction (Burre et al., 2010; Greten-Harrison et al., 2010). Therefore, mouse models of altered α-synuclein expression have increased our understanding of the role of α-synuclein in PD related processes.

### Leucine-Rick Repeat Kinase 2

Missense mutations in the leucine-rich repeat kinase (LRRK2) gene have been identified as causes of autosomal dominant PD with characteristics similar to the sporadic disease forms (Singleton, 2005; Cookson and van der Brug, 2008). LRKK2 is a 2527- amino acid protein consisting of several functional domains, including a leucine-rich repeat domain, a Ras-like small GTPases domain, and a kinase domain with sequence homology to MAP kinase (Tong and Shen, 2009). KO models have been generated for LRRK2 by flanking the first loxP site at intron 1 followed by, a neomycin expression cassette, and the second loxP at intron 2 of the LRRK2 gene (Parisiadou et al., 2009). These KO mice, resulting from crossing the floxed mice with EIIa-Cre-transgenic mice, were viable to adulthood and did not show any major abnormalities, suggesting that LRRK2 may not play a major role in the development and survival of dopaminergic neurons (Andres-Mateos et al., 2009; Parisiadou et al., 2009; Li et al., 2011). Recent studies have shown that the LRRK2 KO mice show increased protein kinase A- mediated phosphorylation of cofilin and glutamate receptor 1 (GluA1) and impaired synaptic transmission in the striatum (Parisiadou et al., 2014). Associations of LRRK2 have been found with microtubule dynamics, in particular the increased levels of soluble β-tubulin in LRRK2 KO mice (Gillardon, 2009). To date, there is no genetic support for the causal involvement of LRRK1, the closest paralog of LRRK2, in PD. Although, LRRK1 and LRRK2 share a high sequence homology in the brain, they function independently through interactions with different cellular proteins (Reyniers et al., 2014). LRRK1/LRRK2 double KO mice are available through Jackson Laboratories, but have not been characterized. Initial linkage studies have identified various LRRK2 missense mutations including G2019S and R1441G in patients with PD (Chen et al., 2012; Herzig et al., 2012). LRRK2 overexpression mouse models were created that bear the inducible G2019S mutation at the conserved Mg^2+^ binding motif within the kinase domain and resulted in consistently enhanced LRRK2 acitivty (Gilks et al., 2005; Nichols et al., 2005; West et al., 2005; Lin et al., 2009). This familial PD-related mutation caused impairments in dopaminergic neurotransmission that was linked to modified localization and phosphorylation of the microtubule-binding protein tau and consequences in motor function (Li et al., 2010; Melrose et al., 2010; Winner et al., 2011). Mice harboring the R1441G mutation demonstrate a strong behavioral phenotype and altered dopamine release recapitulating the cardinal features of PD (Li et al., 2009). In particular these mice showed reduced rearing by 12 months that could be rescued by L-DOPA suggesting that the behaviors are dependent on dopamine. In a more recent study, R1441G KI mice showed perturbed dopamine homeostasis resulting in presynaptic dysfunction and locomotor deficits (Liu et al., 2014). Thus, LRRK2 mouse models have been shown to be a useful tool to understand the role of dopaminergic dysfunction in PD.

### Parkin, Phosphatase and Tensin Homolog (PTEN)-Induced Putative Kinase-1 and DJ-1

Other studies indicate that autosomal recessive forms of familial PD are due to gene mutations in parkin, PTEN-induced putative kinase 1 (PINK1), and DJ-1 (Bonifati et al., 2003b). Parkin is an ubiquitin E3 ligase that is important in adenosine triphosphate-dependent protein degradation (Zhang et al., 2010). Parkin KO mice, with exon 2 replaced with the neomycin-resistant gene, show mild, progressive motor deficits but normal emotionality, learning and memory (Perez and Palmiter, 2005; Harvey et al., 2008). Other parkin KO models have showed that the deletion of exon 3 resulted in reduced synaptic excitability in the nigrostriatal pathway and severe motor impairments in the beam traversal and rotarod tasks (Goldberg et al., 2003). Recent studies indicated that mice with the parkin gene deletion on exon 3 have normal olfaction, anxiety, and depressive-like behaviors, but short-term spatial memory deficits (Rial et al., 2014). However, in all of these KO models, the progressive loss of nigrostriatal dopaminergic neurons or enhanced vulnerability to dopaminergic neurotoxins were not observed (Rial et al., 2014). This suggests that these KO models may be only useful in studying the early-onset stages of PD. Other studies have created transgenic mice expressing a truncated human mutant parkin (Q311 X) driven by a Slc6a3 dopamine transporter promoter (Lu et al., 2009). The parkin-Q311X mice developed age-dependent dopaminergic neuron degeneration in the substantia nigra and showed late-onset and progressive motor deficits (Lu et al., 2009). This study provides evidence for dominant-negative modulatory effects of a parkin mutant on dopaminergic neuron degeneration and hypokinetic motor deficits in PD. Parkin selectively binds to PINK1 and upregulates PINK1 levels, which affects the stability, solubility, and formation of Lewy bodies (Um et al., 2009). PINK1 is a mitochondrial serine/threonine kinase, implicated in apoptosis, mitochondrial dysfunction, and impaired dopamine release (Gandhi et al., 2006). One of the first studies to generate conditional PINK1-silenced transgenic mice used the Cre/loxP inducible system to regulate the expression of PINK1 shRNA by crossing it to CMV-Cre transgenic mice (Zhou et al., 2007). However, these mice showed no striatal dopaminergic neurodegeneration or changes in nigral dopaminergic neuron numbers, and no impairments in motor activity (Zhou et al., 2007) suggesting that the silencing of PINK1 by conditional RNA interference is insufficient to cause dopaminergic neuron death salient with PD. In another study, PINK1 KO mice were created by deleting the kinase domain through introducing a nonsense mutation (Kitada et al., 2007). These KO mice had a normal number of nigral dopaminergic neurons but impairments in corticostriatal LTP and LTD that could be rescued with dopamine receptor agonists (Kitada et al., 2007). Thus, the full rescue of the synaptic deficits indicates that post-synaptic D1 and D2 receptor function is intact and suggest specific presynaptic dopaminergic defects and loss of dopaminergic terminals (Kessler et al., 2005). The precise function of DJ-1 is unknown, however, it has been found to be important in protecting mitochondria against oxidative stress, cellular transformation, transcriptional regulation, and androgen-receptor signaling (Lev et al., 2007). The DJ-1 gene encodes a ubiquitous, highly conserved protein predominantly expressed in neurons and glia cells (Canet-Aviles et al., 2004). Human studies have shown that deletion of the first five exons of the DJ-1 promoter result in progressive neurodegeneration (Bonifati et al., 2003a,b). Thus DJ-1 null mice were generated, lacking the first five exons and part of the promoter region of DJ-1, to mimic the human deletion (Chen et al., 2005). These mice showed age-dependent progression of motor deficits and significantly increased levels of striatal dopamine (Chen et al., 2005). Other studies have examined DJ-1 KO mice through disrupting exon 2, the first coding exon of DJ-1 (Kim et al., 2005). These mice had no changes in nigrostriatal dopamine levels but decreased motor functions (Kim et al., 2005). Together these studies suggest that the loss of parkin, PINK1, or DJ-1 induces motor impairments consistent with the PD phenotype, though their effect on striatal dopaminergic degeneration is inconclusive. Therefore, recent studies have examined the synergistic effects of inactivating all three recessive PD genes that are believed to be essential for the survival of nigral neurons. These triple parkin, PINK1, and DJ-1 KO mice (TKO) were generated by first crossing parkin KO with DJ-1 KO mice, which were then interbred to obtain double parkin/DJ-1 KO mice (Kitada et al., 2009). PINK heterozygous mice were then crossed with parkin KO/DJ-1 heterozygous mice to obtain TKOs. The TKO mice were viable and fertile with no overt brain anatomical abnormalities (Kitada et al., 2009). Surprisingly, these TKO mice showed normal morphology and did not result in a loss of dopaminergic neurons in the substantia nigra and locus coeruleus at ages ranging from 3, 16, 24 months (Kitada et al., 2009). This suggests that all three recessive genes are not required for the survival of dopaminergic neurons but may be protective against age-dependent disruptions in PD.

## Conclusion

In this review, we have briefly discussed selected mouse models of a number of brain disorders that are generated using genetic manipulation techniques, including transgenic mice and knockout/knock-in plus conditional Cre/loxP and inducible systems. The brain diseases that we have focused on include childhood developmental disorders such as autism, ID, FXS, and Williams-Beuren syndrome, neuropsychiatric disorders such as schizophrenia, and neurodegenerative disorders such as AD and PD. It is clear that availability of these mouse models has enabled systematic investigations of the *in vivo* function of many disease-related genes at various levels from gene to behavior, and thus contributed significantly to our understanding of the neurobiological basis of the diseases. It is expected that further analysis of these existing mice, plus additional models with more precise spatial and temporal specificity, will continue to reveal new findings that will enhance our understanding and treatment of various brain diseases.

## Author Contributions

All authors listed, have made substantial, direct and intellectual contribution to the work, and approved it for publication.

## Conflict of Interest Statement

The authors declare that the research was conducted in the absence of any commercial or financial relationships that could be construed as a potential conflict of interest.

The handling Editor declared a shared affiliation, though no other collaboration, with the authors and states that the process nevertheless met the standards of a fair and objective review.

## References

[B1] Abazyan S. Pletnikov M. V. (2014). “Schizophrenia,” in *Behavioural Genetics of the Mouse: Genetic Mouse Models of Neurobehavioral Disorders* eds Pietropaolo F. S. S. Crusio W. E. (Cambridge: Cambridge University Press) 189–207.

[B2] Ahn T. B. Kim S. Y. Kim J. Y. Park S. S. Lee D. S. Min H. J. (2008). alpha-Synuclein gene duplication is present in sporadic Parkinson disease. *Neurology* 70 43–49. 10.1212/01.wnl.0000271080.53272.c7 17625105

[B3] Allen K. M. Gleeson J. G. Bagrodia S. Partington M. W. MacMillan J. C. Cerione R. A. (1998). PAK3 mutation in nonsyndromic X-linked mental retardation. *Nat. Genet.* 20 25–30. 10.1038/1675 9731525

[B4] Amiri A. Sanchez-Ortiz E. Cho W. Birnbaum S. G. Xu J. McKay R. M. (2014). Analysis of FMR1 deletion in a subpopulation of post-mitotic neurons in mouse cortex and hippocampus. *Autism Res.* 7 60–71. 10.1002/aur.1342 24408886

[B5] Anderson G. R. Galfin T. Xu W. Aoto J. Malenka R. C. Sudhof T. C. (2012). Candidate autism gene screen identifies critical role for cell-adhesion molecule CASPR2 in dendritic arborization and spine development. *Proc. Natl. Acad. Sci. U.S.A.* 109 18120–18125. 10.1073/pnas.1216398109 23074245 PMC3497786

[B6] Andres-Mateos E. Mejias R. Sasaki M. Li X. Lin B. M. Biskup S. (2009). Unexpected lack of hypersensitivity in LRRK2 knock-out mice to MPTP (1-methyl-4-phenyl-1,2,3,6-tetrahydropyridine). *J. Neurosci.* 29 15846–15850. 10.1523/JNEUROSCI.4357-09.2009 20016100 PMC2846613

[B7] Arber S. Barbayannis F. A. Hanser H. Schneider C. Stanyon C. A. Bernard O. (1998). Regulation of actin dynamics through phosphorylation of cofilin by LIM-kinase. *Nature* 393 805–809. 10.1038/31729 9655397

[B8] Arendash G. W. King D. L. Gordon M. N. Morgan D. Hatcher J. M. Hope C. E. (2001). Progressive, age-related behavioral impairments in transgenic mice carrying both mutant amyloid precursor protein and presenilin-1 transgenes. *Brain Res.* 891 42–53. 10.1016/S0006-8993(00)03186-3 11164808

[B9] Arking D. E. Cutler D. J. Brune C. W. Teslovich T. M. West K. Ikeda M. (2008). A common genetic variant in the neurexin superfamily member CNTNAP2 increases familial risk of autism. *Am. J. Hum. Genet.* 82 160–164. 10.1016/j.ajhg.2007.09.015 18179894 PMC2253968

[B10] Arnold S. E. Hyman B. T. Flory J. Damasio A. R. Van Hoesen G. W. (1991). The topographical and neuroanatomical distribution of neurofibrillary tangles and neuritic plaques in the cerebral cortex of patients with Alzheimer’s disease. *Cereb. Cortex* 1 103–116. 10.1093/cercor/1.1.103 1822725

[B11] Ascano M. Jr. Mukherjee N. Bandaru P. Miller J. B. Nusbaum J. D. Corcoran D. L. (2012). FMRP targets distinct mRNA sequence elements to regulate protein expression. *Nature* 492 382–386. 10.1038/nature11737 23235829 PMC3528815

[B12] Asrar S. Meng Y. Zhou Z. Todorovski Z. Huang W. W. Jia Z. (2009). Regulation of hippocampal long-term potentiation by p21-activated protein kinase 1 (PAK1). *Neuropharmacology* 56 73–80. 10.1016/j.neuropharm.2008.06.055 18644395

[B13] Austin C. P. Ky B. Ma L. Morris J. A. Shughrue P. J. (2004). Expression of disrupted-in-schizophrenia-1, a schizophrenia-associated gene, is prominent in the mouse hippocampus throughout brain development. *Neuroscience* 124 3–10. 10.1016/j.neuroscience.2003.11.010 14960334

[B14] Autism Genome Project C. Szatmari P. Paterson A. D. Zwaigenbaum L. Roberts W. Brian J. (2007). Mapping autism risk loci using genetic linkage and chromosomal rearrangements. *Nat. Genet.* 39 319–328. 10.1038/ng1985 17322880 PMC4867008

[B15] Ayalew M. Le-Niculescu H. Levey D. F. Jain N. Changala B. Patel S. D. (2012). Convergent functional genomics of schizophrenia: from comprehensive understanding to genetic risk prediction. *Mol. Psychiatry* 17 887–905. 10.1038/mp.2012.37 22584867 PMC3427857

[B16] Bakker C. E. Verheij C. Willemsen R. Vanderhelm R. Oerlemans F. Vermey M. (1994). Fmr1 knockout mice: a model to study fragile X mental retardation. *Cell* 78 23–33. 8033209

[B17] Baskaran Y. Ng Y. W. Selamat W. Ling F. T. Manser E. (2012). Group I and II mammalian PAKs have different modes of activation by Cdc42. *EMBO Rep.* 13 653–659. 10.1038/embor.2012.75 22653441 PMC3388789

[B18] Basuta K. Narcisa V. Chavez A. Kumar M. Gane L. Hagerman R. (2011). Clinical phenotypes of a juvenile sibling pair carrying the fragile X premutation. *Am. J. Med. Genet. A* 155A 519–525. 10.1002/ajmg.a.33446 21344625 PMC3568664

[B19] Bayer T. A. Cappai R. Masters C. L. Beyreuther K. Multhaup G. (1999). It all sticks together–the APP-related family of proteins and Alzheimer’s disease. *Mol. Psychiatry* 4 524–528. 10.1038/sj.mp.4000552 10578233

[B20] Berry-Kravis E. Abrams L. Coffey S. M. Hall D. A. Greco C. Gane L. W. (2007). Fragile X-associated tremor/ataxia syndrome: clinical features, genetics, and testing guidelines. *Mov. Disord.* 22 2018–2030. 10.1002/mds.21493 17618523

[B21] Blundell J. Blaiss C. A. Etherton M. R. Espinosa F. Tabuchi K. Walz C. (2010). Neuroligin-1 deletion results in impaired spatial memory and increased repetitive behavior. *J. Neurosci.* 30 2115–2129. 10.1523/JNEUROSCI.4517-09.2010 20147539 PMC2824441

[B22] Bockers T. M. Mameza M. G. Kreutz M. R. Bockmann J. Weise C. Buck F. (2001). Synaptic scaffolding proteins in rat brain. Ankyrin repeats of the multidomain Shank protein family interact with the cytoskeletal protein alpha-fodrin. *J. Biol. Chem.* 276 40104–40112. 10.1074/jbc.M102454200 11509555

[B23] Boda B. Alberi S. Nikonenko I. Node-Langlois R. Jourdain P. Moosmayer M. (2004). The mental retardation protein PAK3 contributes to synapse formation and plasticity in hippocampus. *J. Neurosci.* 24 10816–10825. 10.1523/JNEUROSCI.2931-04.2004 15574732 PMC6730202

[B24] Bolivar V. J. (2014). “Social dysfunction and mental retardation- autism,” in *Behavioral Genetics of the Mouse: Genetic Mouse Models of Neurobehavioural Disorders* eds Pietropaolo S. Sluyter F. Crusio W. E. (Cambridge: Cambridge University Press) 113–133.

[B25] Bonifati V. Rizzu P. Squitieri F. Krieger E. Vanacore N. van Swieten J. C. (2003a). DJ-1(PARK7), a novel gene for autosomal recessive, early onset parkinsonism. *Neurol. Sci.* 24 159–160. 10.1007/s10072-003-0108-0 14598065

[B26] Bonifati V. Rizzu P. van Baren M. J. Schaap O. Breedveld G. J. Krieger E. (2003b). Mutations in the DJ-1 gene associated with autosomal recessive early-onset parkinsonism. *Science* 299 256–259. 10.1126/science.1077209 12446870

[B27] Bontekoe C. J. McIlwain K. L. Nieuwenhuizen I. M. Yuva-Paylor L. A. Nellis A. Willemsen R. (2002). Knockout mouse model for Fxr2: a model for mental retardation. *Hum. Mol. Genet.* 11 487–498. 10.1093/hmg/11.5.487 11875043

[B28] Borchelt D. R. Thinakaran G. Eckman C. B. Lee M. K. Davenport F. Ratovitsky T. (1996). Familial Alzheimer’s disease-linked presenilin 1 variants elevate Abeta1-42/1-40 ratio in vitro and in vivo. *Neuron* 17 1005–1013. 10.1016/S0896-6273(00)80230-5 8938131

[B29] Bozdagi O. Sakurai T. Papapetrou D. Wang X. Dickstein D. L. Takahashi N. (2010). Haploinsufficiency of the autism-associated Shank3 gene leads to deficits in synaptic function, social interaction, and social communication. *Mol. Autism* 1:15 10.1186/2040-2392-1-15 PMC3019144 21167025

[B30] Braak H. Braak E. (1994). Morphological criteria for the recognition of Alzheimer’s disease and the distribution pattern of cortical changes related to this disorder. *Neurobiol. Aging* 15 355–356 discussion 379–380 10.1016/0197-4580(94)90032-9 7936061

[B31] Braak H. Del Tredici K. Rub U. de Vos R. A. Jansen Steur E. N. Braak E. (2003). Staging of brain pathology related to sporadic Parkinson’s disease. *Neurobiol. Aging* 24 197–211. 10.1016/S0197-4580(02)00065-9 12498954

[B32] Brandon N. J. Millar J. K. Korth C. Sive H. Singh K. K. Sawa A. (2009). Understanding the role of DISC1 in psychiatric disease and during normal development. *J. Neurosci.* 29 12768–12775. 10.1523/JNEUROSCI.3355-09.2009 19828788 PMC6665304

[B33] Bregman J. D. Leckman J. F. Ort S. I. (1988). Fragile X syndrome: genetic predisposition to psychopathology. *J. Autism. Dev. Disord.* 18 343–354. 10.1007/BF02212191 3170453

[B34] Brennan F. X. Albeck D. S. Paylor R. (2006). Fmr1 knockout mice are impaired in a leverpress escape/avoidance task. *Genes Brain Behav.* 5 467–471. 10.1111/j.1601-183X.2005.00183.x 16923151

[B35] Britsch S. (2007). The neuregulin-I/ErbB signaling system in development and disease. *Adv. Anat. Embryol. Cell Biol.* 190 1–65. 17432114

[B36] Burden S. Yarden Y. (1997). Neuregulins and their receptors: a versatile signaling module in organogenesis and oncogenesis. *Neuron* 18 847–855. 10.1016/S0896-6273(00)80324-4 9208852

[B37] Burre J. Sharma M. Tsetsenis T. Buchman V. Etherton M. R. Sudhof T. C. (2010). Alpha-synuclein promotes SNARE-complex assembly in vivo and in vitro. *Science* 329 1663–1667. 10.1126/science.1195227 20798282 PMC3235365

[B38] Calhoun M. E. Wiederhold K. H. Abramowski D. Phinney A. L. Probst A. Sturchler-Pierrat C. (1998). Neuron loss in APP transgenic mice. *Nature* 395 755–756. 10.1038/27351 9796810

[B39] Camargo L. M. Collura V. Rain J. C. Mizuguchi K. Hermjakob H. Kerrien S. (2007). Disrupted in schizophrenia 1 interactome: evidence for the close connectivity of risk genes and a potential synaptic basis for schizophrenia. *Mol. Psychiatry* 12 74–86. 10.1038/sj.mp.4001880 17043677

[B40] Canet-Aviles R. M. Wilson M. A. Miller D. W. Ahmad R. McLendon C. Bandyopadhyay S. (2004). The Parkinson’s disease protein DJ-1 is neuroprotective due to cysteine-sulfinic acid-driven mitochondrial localization. *Proc. Natl. Acad. Sci. U.S.A.* 101 9103–9108. 10.1073/pnas.0402959101 15181200 PMC428480

[B41] Carlsson A. Lindqvist M. (1963). Effect of chlorpromazine or haloperidol on formation of 3methoxytyramine and normetanephrine in mouse brain. *Acta Pharmacol. Toxicol. (Copenh)* 20 140–144. 10.1111/j.1600-0773.1963.tb01730.x 14060771

[B42] Chandra S. Fornai F. Kwon H. B. Yazdani U. Atasoy D. Liu X. (2004). Double-knockout mice for alpha- and beta-synucleins: effect on synaptic functions. *Proc. Natl. Acad. Sci. U.S.A.* 101 14966–14971. 10.1073/pnas.0406283101 15465911 PMC522043

[B43] Chandra S. Gallardo G. Fernandex-Chancon R. Schluter O. M. Sudhof T. C. (2005). alpha-synuclein cooperates with CSPalpha in preventing neurodegeneration. *Cell* 123 383–396. 10.1016/j.cell.2005.09.028 16269331

[B44] Chandran J. S. Kazanis I. Clapcote S. J. Ogawa F. Millar J. K. Porteous D. J. (2014). Disc1 variation leads to specific alterations in adult neurogenesis. *PLoS ONE* 9:e108088 10.1371/journal.pone.0108088 PMC4182707 25272038

[B45] Chelly J. Mandel J. L. (2001). Monogenic causes of X-linked mental retardation. *Nat. Rev. Genet.* 2 669–680. 10.1038/35088558 11533716

[B46] Chen C. Y. Weng Y. H. Chien K. Y. Lin K. J. Yeh T. H. Cheng Y. P. (2012). (G2019S) LRRK2 activates MKK4-JNK pathway and causes degeneration of SN dopaminergic neurons in a transgenic mouse model of PD. *Cell Death. Differ.* 19 1623–1633. 10.1038/cdd.2012.42 22539006 PMC3438494

[B47] Chen L. Cagniard B. Mathews T. Jones S. Koh H. C. Ding Y. (2005). Age-dependent motor deficits and dopaminergic dysfunction in DJ-1 null mice. *J. Biol. Chem.* 280 21418–21426. 10.1074/jbc.M413955200 15799973

[B48] Chonchaiya W. Au J. Schneider A. Hessl D. Harris S. W. Laird M. (2012). Increased prevalence of seizures in boys who were probands with the FMR1 premutation and co-morbid autism spectrum disorder. *Hum. Genet.* 131 581–589. 10.1007/s00439-011-1106-6 22001913 PMC4105134

[B49] Chubb J. E. Bradshaw N. J. Soares D. C. Porteous D. J. Millar J. K. (2008). The DISC locus in psychiatric illness. *Mol. Psychiatry* 13 36–64. 10.1038/sj.mp.4002106 17912248

[B50] Clapcote S. J. Lipina T. V. Millar J. K. Mackie S. Christie S. Ogawa F. (2007). Behavioral phenotypes of Disc1 missense mutations in mice. *Neuron* 54 387–402. 10.1016/j.neuron.2007.04.015 17481393

[B51] Clark L. N. Poorkaj P. Wszolek Z. Geschwind D. H. Nasreddine Z. S. Miller B. (1998). Pathogenic implications of mutations in the tau gene in pallido-ponto-nigral degeneration and related neurodegenerative disorders linked to chromosome 17. *Proc. Natl. Acad. Sci. U.S.A.* 95 13103–13107. 10.1073/pnas.95.22.13103 9789048 PMC23724

[B52] Coghill E. L. Hugill A. Parkinson N. Davison C. Glenister P. Clements S. (2002). A gene-driven approach to the identification of ENU mutants in the mouse. *Nat. Genet.* 30 255–256. 10.1038/ng847 11850622

[B53] Comery T. A. Martone R. L. Aschmies S. Atchison K. P. Diamantidis G. Gong X. (2005). Acute gamma-secretase inhibition improves contextual fear conditioning in the Tg2576 mouse model of Alzheimer’s disease. *J. Neurosci.* 25 8898–8902. 10.1523/JNEUROSCI.2693-05.2005 16192379 PMC6725598

[B54] Cookson M. R. van der Brug M. (2008). Cell systems and the toxic mechanism(s) of alpha-synuclein. *Exp. Neurol.* 209 5–11. 10.1016/j.expneurol.2007.05.022 17603039 PMC2231843

[B55] Coyle J. T. (2006). Substance use disorders and Schizophrenia: a question of shared glutamatergic mechanisms. *Neurotox. Res.* 10 221–233. 10.1007/BF03033359 17197372

[B56] Crawford D. C. Acuna J. M. Sherman S. L. (2001). FMR1 and the fragile X syndrome: human genome epidemiology review. *Genet. Med.* 3 359–371. 10.1097/00125817-200109000-00006 11545690 PMC4493892

[B57] Dachtler J. Ivorra J. L. Rowland T. E. Lever C. Rodgers R. J. Clapcote S. J. (2015). Heterozygous deletion of alpha-neurexin I or alpha-neurexin II results in behaviors relevant to autism and schizophrenia. *Behav. Neurosci.* 129 765–776. 10.1037/bne0000108 26595880 PMC4655861

[B58] Dahlhaus R. Hines R. M. Eadie B. D. Kannangara T. S. Hines D. J. Brown C. E. (2010). Overexpression of the cell adhesion protein neuroligin-1 induces learning deficits and impairs synaptic plasticity by altering the ratio of excitation to inhibition in the hippocampus. *Hippocampus* 20 305–322. 10.1002/hipo.20630 19437420

[B59] Davis K. L. Kahn R. S. Ko G. Davidson M. (1991). Dopamine in schizophrenia: a review and reconceptualization. *Am. J. Psychiatry* 148 1474–1486. 10.1176/ajp.148.11.1474 1681750

[B60] Dawson H. N. Ferreira A. Eyster M. V. Ghoshal N. Binder L. I. Vitek M. P. (2001). Inhibition of neuronal maturation in primary hippocampal neurons from tau deficient mice. *J. Cell Sci.* 114 1179–1187. 11228161 10.1242/jcs.114.6.1179

[B61] De Strooper B. (2007). Loss-of-function presenilin mutations in Alzheimer disease. Talking Point on the role of presenilin mutations in Alzheimer disease. *EMBO Rep.* 8 141–146. 10.1038/sj.embor.7400897 17268505 PMC1796779

[B62] DesMarais V. Ghosh M. Eddy R. Condeelis J. (2005). Cofilin takes the lead. *J. Cell Sci.* 118 19–26. 10.1242/jcs.01631 15615780

[B63] Devys D. Lutz Y. Rouyer N. Bellocq J. P. Mandel J. L. (1993). The FMR-1 protein is cytoplasmic, most abundant in neurons and appears normal in carriers of a fragile X premutation. *Nat. Genet.* 4 335–340. 10.1038/ng0893-335 8401578

[B64] Dodart J. C. Meziane H. Mathis C. Bales K. R. Paul S. M. Ungerer A. (1999). Behavioral disturbances in transgenic mice overexpressing the V717F beta-amyloid precursor protein. *Behav. Neurosci.* 113 982–990. 10.1037/0735-7044.113.5.982 10571480

[B65] Drolet R. E. Behrouz B. Lookingland K. J. Goudreau J. L. (2004). Mice lacking alpha-synuclein have an attenuated loss of striatal dopamine following prolonged chronic MPTP administration. *Neurotoxicology* 25 761–769. 10.1016/j.neuro.2004.05.002 15288507

[B66] Dubos A. Combeau G. Bernardinelli Y. Barnier J. V. Hartley O. Gaertner H. (2012). Alteration of synaptic network dynamics by the intellectual disability protein PAK3. *J. Neurosci.* 32 519–527. 10.1523/JNEUROSCI.3252-11.2012 22238087 PMC6621069

[B67] Duffy L. Cappas E. Scimone A. Schofield P. R. Karl T. (2008). Behavioral profile of a heterozygous mutant mouse model for EGF-like domain neuregulin 1. *Behav. Neurosci.* 122 748–759. 10.1037/0735-7044.122.4.748 18729627

[B68] Duncan G. Miyamoto S. Gu H. Lieberman J. Koller B. Snouwaert J. (2002). Alterations in regional brain metabolism in genetic and pharmacological models of reduced NMDA receptor function. *Brain Res.* 951 166–176. 10.1016/S0006-8993(02)03156-6 12270494

[B69] Durand C. M. Betancur C. Boeckers T. M. Bockmann J. Chaste P. Fauchereau F. (2007). Mutations in the gene encoding the synaptic scaffolding protein SHANK3 are associated with autism spectrum disorders. *Nat. Genet.* 39 25–27. 10.1038/ng1933 17173049 PMC2082049

[B70] Durieux A. M. Horder J. Petrinovic M. M. (2015). Neuroligin-2 and the tightrope of excitation/inhibition balance in the prefrontal cortex. *J. Neurophysiol.* 115 5–7. 10.1152/jn.00703.2015 26224778 PMC4760485

[B71] Eberhard D. Grummt I. (1996). Species specificity of ribosomal gene transcription: a factor associated with human RNA polymerase I prevents transcription of mouse rDNA. *DNA Cell Biol.* 15 167–173. 10.1089/dna.1996.15.167 8634144

[B72] Elder G. A. Gama Sosa M. A. De Gasperi R. Dickstein D. L. Hof P. R. (2010). Presenilin transgenic mice as models of Alzheimer’s disease. *Brain Struct. Funct.* 214 127–143. 10.1007/s00429-009-0227-3 19921519 PMC3527905

[B73] Etherton M. R. Blaiss C. A. Powell C. M. Sudhof T. C. (2009). Mouse neurexin-1alpha deletion causes correlated electrophysiological and behavioral changes consistent with cognitive impairments. *Proc. Natl. Acad. Sci. U.S.A.* 106 17998–18003. 10.1073/pnas.0910297106 19822762 PMC2764944

[B74] Ey E. Yang M. Katz A. M. Woldeyohannes L. Silverman J. L. Leblond C. S. (2012). Absence of deficits in social behaviors and ultrasonic vocalizations in later generations of mice lacking neuroligin4. *Genes Brain Behav.* 11 928–941. 10.1111/j.1601-183X.2012.00849.x 22989184 PMC4904774

[B75] Fearnley J. M. Lees A. J. (1991). Ageing and Parkinson’s disease: substantia nigra regional selectivity. *Brain* 114(Pt 5) 2283–2301. 10.1093/brain/114.5.2283 1933245

[B76] Feng R. Wang H. Wang J. Shrom D. Zheng X. Tsien J. Z. (2004). Forebrain degeneration and ventricle enlargement caused by double knockout of Alzheimer’s presenilin-1 and presenilin-2. *Proc. Natl. Acad. Sci. U.S.A.* 101 8162–8167. 10.1073/pnas.0402733101 15148382 PMC419574

[B77] Field J. Manser E. (2012). The PAKs come of age: celebrating 18 years of discovery. *Cell. Logist.* 2 54–58. 10.4161/cl.22084 23125949 PMC3485743

[B78] Foldy C. Malenka R. C. Sudhof T. C. (2013). Autism-associated neuroligin-3 mutations commonly disrupt tonic endocannabinoid signaling. *Neuron* 78 498–509. 10.1016/j.neuron.2013.02.036 23583622 PMC3663050

[B79] Forno L. S. (1996). Neuropathology of Parkinson’s disease. *J. Neuropathol. Exp. Neurol.* 55 259–272. 10.1097/00005072-199603000-00001 8786384

[B80] Frangiskakis J. M. Ewart A. K. Morris C. A. Mervis C. B. Bertrand J. Robinson B. F. (1996). *LIM-kinase1* hemizygosity implicated in impaired visuospatial constructive cognition. *Cell* 86 59–69. 10.1016/S0092-8674(00)80077-X 8689688

[B81] Fridell R. A. Benson R. E. Hua J. Bogerd H. P. Cullen B. R. (1996). A nuclear role for the Fragile X mental retardation protein. *EMBO J.* 15 5408–5414. 8895584 PMC452283

[B82] Fu Y. H. Kuhl D. P. Pizzuti A. Pieretti M. Sutcliffe J. S. Richards S. (1991). Variation of the CGG repeat at the fragile X site results in genetic instability: resolution of the Sherman paradox. *Cell* 67 1047–1058. 10.1016/0092-8674(91)90283-5 1760838

[B83] Games D. Adams D. Alessandrini R. Barbour R. Berthelette P. Blackwell C. (1995). Alzheimer-type neuropathology in transgenic mice overexpressing V717F beta-amyloid precursor protein. *Nature* 373 523–527. 10.1038/373523a0 7845465

[B84] Gandhi S. Muqit M. M. Stanyer L. Healy D. G. Abou-Sleiman P. M. Hargreaves I. (2006). PINK1 protein in normal human brain and Parkinson’s disease. *Brain* 129 1720–1731. 10.1093/brain/awl114 16702191

[B85] Gasser T. (2009). Genomic and proteomic biomarkers for Parkinson disease. *Neurology* 72 S27–S31. 10.1212/WNL.0b013e318198e054 19221311

[B86] Gdalyahu A. Lazaro M. Penagarikano O. Golshani P. Trachtenberg J. T. Geschwind D. H. (2015). The autism related protein contactin-associated protein-like 2 (CNTNAP2) stabilizes new spines: an in vivo mouse study. *PLoS ONE* 10:e0125633 10.1371/journal.pone.0125633 PMC4423902 25951243

[B87] Gerlai R. Pisacane P. Erickson S. (2000). Heregulin, but not ErbB2 or ErbB3, heterozygous mutant mice exhibit hyperactivity in multiple behavioral tasks. *Behav. Brain Res.* 109 219–227. 10.1016/S0166-4328(99)00175-8 10762692

[B88] Gilks W. P. Abou-Sleiman P. M. Gandhi S. Jain S. Singleton A. Lees A. J. (2005). A common LRRK2 mutation in idiopathic Parkinson’s disease. *Lancet* 365 415–416. 10.1016/S0140-6736(05)17830-1 15680457

[B89] Gillardon F. (2009). Leucine-rich repeat kinase 2 phosphorylates brain tubulin-beta isoforms and modulates microtubule stability–a point of convergence in parkinsonian neurodegeneration? *J. Neurochem.* 110 1514–1522. 10.1111/j.1471-4159.2009.06235.x 19545277

[B90] Glessner J. T. Wang K. Cai G. Korvatska O. Kim C. E. Wood S. (2009). Autism genome-wide copy number variation reveals ubiquitin and neuronal genes. *Nature* 459 569–573. 10.1038/nature07953 19404257 PMC2925224

[B91] Goate A. Chartier-Harlin M. C. Mullan M. Brown J. Crawford F. Fidani L. (1991). Segregation of a missense mutation in the amyloid precursor protein gene with familial Alzheimer’s disease. *Nature* 349 704–706. 10.1038/349704a0 1671712

[B92] Goedert M. (2005). Tau gene mutations and their effects. *Mov. Disord.* 20(Suppl. 12) S45–S52. 10.1002/mds.20539 16092090

[B93] Goldberg M. S. Fleming S. M. Palacino J. J. Cepeda C. Lam H. A. Bhatnagar A. (2003). Parkin-deficient mice exhibit nigrostriatal deficits but not loss of dopaminergic neurons. *J. Biol. Chem.* 278 43628–43635. 10.1074/jbc.M308947200 12930822

[B94] Gordon A. Adamsky K. Vainshtein A. Frechter S. Dupree J. L. Rosenbluth J. (2014). Caspr and caspr2 are required for both radial and longitudinal organization of myelinated axons. *J. Neurosci.* 34 14820–14826. 10.1523/JNEUROSCI.3369-14.2014 25378149 PMC4220019

[B95] Greten-Harrison B. Polydoro M. Morimoto-Tomita M. Diao L. Williams A. M. Nie E. H. (2010). alphabetagamma-synuclein triple knockout mice reveal age-dependent neuronal dysfunction. *Proc. Natl. Acad. Sci. U.S.A.* 107 19573–19578. 10.1073/pnas.1005005107 20974939 PMC2984188

[B96] Hall A. M. Roberson E. D. (2012). Mouse models of Alzheimer’s disease. *Brain Res. Bull.* 88 3–12. 10.1016/j.brainresbull.2011.11.017 22142973 PMC3546481

[B97] Hammer M. Krueger-Burg D. Tuffy L. P. Cooper B. H. Taschenberger H. Goswami S. P. (2015). Perturbed hippocampal synaptic inhibition and gamma-oscillations in a neuroligin-4 knockout mouse model of autism. *Cell Rep.* 13 516–523. 10.1016/j.celrep.2015.09.011 26456829 PMC5862414

[B98] Harada A. Oguchi K. Okabe S. Kuno J. Terada S. Ohshima T. (1994). Altered microtubule organization in small-calibre axons of mice lacking tau protein. *Nature* 369 488–491. 10.1038/369488a0 8202139

[B99] Harrison P. J. Law A. J. (2006). Neuregulin 1 and schizophrenia: genetics, gene expression, and neurobiology. *Biol. Psychiatry* 60 132–140. 10.1016/j.biopsych.2005.11.002 16442083

[B100] Harvey B. K. Wang Y. Hoffer B. J. (2008). Transgenic rodent models of Parkinson’s disease. *Acta Neurochir. Suppl.* 101 89–92. 10.1007/978-3-211-78205-7_15 18642640 PMC2613245

[B101] Hayashi M. L. Choi S. Y. Rao B. S. Jung H. Y. Lee H. K. Zhang D. (2004). Altered cortical synaptic morphology and impaired memory consolidation in forebrain- specific dominant-negative PAK transgenic mice. *Neuron* 42 773–787. 10.1016/j.neuron.2004.05.003 15182717

[B102] Hayashi M. L. Rao B. S. Seo J. S. Choi H. S. Dolan B. M. Choi S. Y. (2007). Inhibition of p21-activated kinase rescues symptoms of fragile X syndrome in mice. *Proc. Natl. Acad. Sci. U.S.A.* 104 11489–11494. 10.1073/pnas.0705003104 17592139 PMC1899186

[B103] Herzig M. C. Bidinosti M. Schweizer T. Hafner T. Stemmelen C. Weiss A. (2012). High LRRK2 levels fail to induce or exacerbate neuronal alpha-synucleinopathy in mouse brain. *PLoS ONE* 7:e36581 10.1371/journal.pone.0036581 PMC3352901 22615783

[B104] Hikida T. Jaaro-Peled H. Seshadri S. Oishi K. Hookway C. Kong S. (2007). Dominant-negative DISC1 transgenic mice display schizophrenia-associated phenotypes detected by measures translatable to humans. *Proc. Natl. Acad. Sci. U.S.A.* 104 14501–14506. 10.1073/pnas.0704774104 17675407 PMC1964873

[B105] Hines R. M. Wu L. Hines D. J. Steenland H. Mansour S. Dahlhaus R. (2008). Synaptic imbalance, stereotypies, and impaired social interactions in mice with altered neuroligin 2 expression. *J. Neurosci.* 28 6055–6067. 10.1523/JNEUROSCI.0032-08.2008 18550748 PMC6670530

[B106] Hinton V. J. Brown W. T. Wisniewski K. Rudelli R. D. (1991). Analysis of neocortex in three males with the fragile X syndrome. *Am. J. Med. Genet.* 41 289–294. 10.1002/ajmg.1320410306 1724112

[B107] Hirtz D. Thurman D. J. Gwinn-Hardy K. Mohamed M. Chaudhuri A. R. Zalutsky R. (2007). How common are the “common” neurologic disorders? *Neurology* 68 326–337. 10.1212/01.wnl.0000252807.38124.a3 17261678

[B108] Holmes W. E. Sliwkowski M. X. Akita R. W. Henzel W. J. Lee J. Park J. W. (1992). Identification of heregulin, a specific activator of p185erbB2. *Science* 256 1205–1210. 10.1126/science.256.5060.1205 1350381

[B109] Hoogenraad C. C. Koekkoek B. Akhmanova A. Krugers H. Dortland B. Miedema M. (2002). Targeted mutation of Cyln2 in the Williams syndrome critical region links CLIP-115 haploinsufficiency to neurodevelopmental abnormalities in mice. *Nat. Genet.* 32 116–127. 10.1038/ng954 12195424

[B110] Howes O. D. Kapur S. (2009). The dopamine hypothesis of schizophrenia: version III–the final common pathway. *Schizophr. Bull.* 35 549–562. 10.1093/schbul/sbp006 19325164 PMC2669582

[B111] Hsiao K. (1998). Transgenic mice expressing Alzheimer amyloid precursor proteins. *Exp. Gerontol.* 33 883–889. 10.1016/S0531-5565(98)00045-X 9951631

[B112] Huang W. Zhou Z. Asrar S. Henkelman M. Xie W. Jia Z. (2011). p21-Activated kinases 1 and 3 control brain size through coordinating neuronal complexity and synaptic properties. *Mol. Cell. Biol.* 31 388–403. 10.1128/MCB.00969-10 21115725 PMC3028630

[B113] Hutton M. Lendon C. L. Rizzu P. Baker M. Froelich S. Houlden H. (1998). Association of missense and 5’-splice-site mutations in tau with the inherited dementia FTDP-17. *Nature* 393 702–705. 10.1038/31508 9641683

[B114] Ichtchenko K. Hata Y. Nguyen T. Ullrich B. Missler M. Moomaw C. (1995). Neuroligin 1: a splice site-specific ligand for beta-neurexins. *Cell* 81 435–443. 10.1016/0092-8674(95)90396-8 7736595

[B115] Ikegami S. Harada A. Hirokawa N. (2000). Muscle weakness, hyperactivity, and impairment in fear conditioning in tau-deficient mice. *Neurosci. Lett.* 279 129–132. 10.1016/S0304-3940(99)00964-7 10688046

[B116] Irwin S. A. Patel B. Idupulapati M. Harris J. B. Crisostomo R. A. Larsen B. P. (2001). Abnormal dendritic spine characteristics in the temporal and visual cortices of patients with fragile-X syndrome: a quantitative examination. *Am. J. Med. Genet.* 98 161–167. 10.1002/1096-8628(20010115)98:2<161::AID-AJMG1025>3.0.CO;2-B 11223852

[B117] Jamain S. Radyushkin K. Hammerschmidt K. Granon S. Boretius S. Varoqueaux F. (2008). Reduced social interaction and ultrasonic communication in a mouse model of monogenic heritable autism. *Proc. Natl. Acad. Sci. U.S.A.* 105 1710–1715. 10.1073/pnas.0711555105 18227507 PMC2234209

[B118] Jankovic J. (2008). Parkinson’s disease: clinical features and diagnosis. *J. Neurol. Neurosurg. Psychiatry* 79 368–376. 10.1136/jnnp.2007.131045 18344392

[B119] Janus C. Borchelt D. (2014). “Alzheimer’s disease,” in *Behavioral Genetics of the Mouse: Genetic Mouse Models of Neurobehavioral Disorders* eds Pietropaolo F. S. S. Crusio W. E. (Cambridge: Cambridge University Press) 391–410.

[B120] Janus C. D’Amelio S. Amitay O. Chishti M. A. Strome R. Fraser P. (2000). Spatial learning in transgenic mice expressing human presenilin 1 (PS1) transgenes. *Neurobiol. Aging* 21 541–549. 10.1016/S0197-4580(00)00107-X 10924767

[B121] Jaramillo T. C. Speed H. E. Xuan Z. Reimers J. M. Liu S. Powell C. M. (2015). Altered striatal synaptic function and abnormal behaviour in shank3 exon4-9 deletion mouse model of autism. *Autism Res.* 10.1002/aur.1529 [Epub ahead of print]. PMC4857590 26559786

[B122] Javitt D. C. (2007). Glutamate and schizophrenia: phencyclidine, N-methyl-D-aspartate receptors, and dopamine-glutamate interactions. *Int. Rev. Neurobiol.* 78 69–108. 10.1016/S0074-7742(06)78003-5 17349858

[B123] Javitt D. C. Zukin S. R. (1991). Recent advances in the phencyclidine model of schizophrenia. *Am. J. Psychiatry* 148 1301–1308. 10.1176/ajp.148.10.1301 1654746

[B124] Jedlicka P. Vnencak M. Krueger D. D. Jungenitz T. Brose N. Schwarzacher S. W. (2013). Neuroligin-1 regulates excitatory synaptic transmission, LTP and EPSP-spike coupling in the dentate gyrus in vivo. *Brain Struct. Funct.* 10.1007/s00429-013-0636-1PMID:NOPMID [Epub ahead of print]. 25713840

[B125] Jia Z. P. Todorovski Z. Meng Y. H. Asrar S. Wang L. Y. (2009). “LIMK-1 and actin regulation of spine and synaptic function,” in *New Encyclopedia of Neuroscience* ed. Squire L. R. (Oxford: Academic Press) 467–472.

[B126] Juan L. W. Liao C. C. Lai W. S. Chang C. Y. Pei J. C. Wong W. R. (2014). Phenotypic characterization of C57BL/6J mice carrying the Disc1 gene from the 129S6/SvEv strain. *Brain Struct. Funct.* 219 1417–1431. 10.1007/s00429-013-0577-8 23689501

[B127] Karl T. Duffy L. Scimone A. Harvey R. P. Schofield P. R. (2007). Altered motor activity, exploration and anxiety in heterozygous neuregulin 1 mutant mice: implications for understanding schizophrenia. *Genes Brain Behav.* 6 677–687. 10.1111/j.1601-183X.2006.00298.x 17309661

[B128] Kato T. Kasai A. Mizuno M. Fengyi L. Shintani N. Maeda S. (2010). Phenotypic characterization of transgenic mice overexpressing neuregulin-1. *PLoS ONE* 5:e14185 10.1371/journal.pone.0014185 PMC3000321 21151609

[B129] Kawarabayashi T. Younkin L. H. Saido T. C. Shoji M. Ashe K. H. Younkin S. G. (2001). Age-dependent changes in brain, CSF, and plasma amyloid (beta) protein in the Tg2576 transgenic mouse model of Alzheimer’s disease. *J. Neurosci.* 21 372–381. 11160418 10.1523/JNEUROSCI.21-02-00372.2001 PMC6763819

[B130] Kellendonk C. Simpson E. H. Polan H. J. Malleret G. Vronskaya S. Winiger V. (2006). Transient and selective overexpression of dopamine D2 receptors in the striatum causes persistent abnormalities in prefrontal cortex functioning. *Neuron* 49 603–615. 10.1016/j.neuron.2006.01.023 16476668

[B131] Kelly M. L. Chernoff J. (2012). Mouse models of PAK function. *Cell Logist.* 2 84–88. 10.4161/cl.21381 23162740 PMC3490966

[B132] Kessler K. R. Hamscho N. Morales B. Menzel C. Barrero F. Vives F. (2005). Dopaminergic function in a family with the PARK6 form of autosomal recessive Parkinson’s syndrome. *J. Neural Transm. (Vienna)* 112 1345–1353. 10.1007/s00702-005-0281-9 15785866

[B133] Kim J. Jung S. Y. Lee Y. K. Park S. Choi J. S. Lee C. J. (2008). Neuroligin-1 is required for normal expression of LTP and associative fear memory in the amygdala of adult animals. *Proc. Natl. Acad. Sci. U.S.A.* 105 9087–9092. 10.1073/pnas.0803448105 18579781 PMC2449369

[B134] Kim R. H. Peters M. Jang Y. Shi W. Pintilie M. Fletcher G. C. (2005). DJ-1, a novel regulator of the tumor suppressor PTEN. *Cancer Cell* 7 263–273. 10.1016/j.ccr.2005.02.010 15766664

[B135] Kirkpatrick L. L. McIlwain K. A. Nelson D. L. (2001). Comparative genomic sequence analysis of the FXR gene family: FMR1, FXR1, and FXR2. *Genomics* 78 169–177. 10.1006/geno.2001.6667 11735223

[B136] Kitada T. Pisani A. Porter D. R. Yamaguchi H. Tscherter A. Martella G. (2007). Impaired dopamine release and synaptic plasticity in the striatum of PINK1-deficient mice. *Proc. Natl. Acad. Sci. U.S.A.* 104 11441–11446. 10.1073/pnas.0702717104 17563363 PMC1890561

[B137] Kitada T. Tong Y. Gautier C. A. Shen J. (2009). Absence of nigral degeneration in aged parkin/DJ-1/PINK1 triple knockout mice. *J. Neurochem.* 111 696–702. 10.1111/j.1471-4159.2009.06350.x 19694908 PMC2952933

[B138] Koekkoek S. K. Yamaguchi K. Milojkovic B. A. Dortland B. R. Ruigrok T. J. Maex R. (2005). Deletion of FMR1 in Purkinje cells enhances parallel fiber LTD, enlarges spines, and attenuates cerebellar eyelid conditioning in Fragile X syndrome. *Neuron* 47 339–352. 10.1016/j.neuron.2005.07.005 16055059

[B139] Koike H. Arguello P. A. Kvajo M. Karayiorgou M. Gogos J. A. (2006). Disc1 is mutated in the 129S6/SvEv strain and modulates working memory in mice. *Proc. Natl. Acad. Sci. U.S.A.* 103 3693–3697. 10.1073/pnas.0511189103 16484369 PMC1450143

[B140] Kooy R. F. D’Hooge R. Reyniers E. Bakker C. E. Nagels G. De Boulle K. (1996). Transgenic mouse model for the fragile X syndrome. *Am. J. Med. Genet.* 64 241–245. 10.1002/(SICI)1096-8628(19960809)64:2<241::AID-AJMG1>3.0.CO;2-X 8844056

[B141] Kremer E. J. Pritchard M. Lynch M. Yu S. Holman K. Baker E. (1991a). Mapping of DNA instability at the fragile X to a trinucleotide repeat sequence p(CCG)n. *Science* 252 1711–1714. 10.1126/science.1675488 1675488

[B142] Kremer E. J. Yu S. Pritchard M. Nagaraja R. Heitz D. Lynch M. (1991b). Isolation of a human DNA sequence which spans the fragile X. *Am. J. Hum. Genet.* 49 656–661. 1882843 PMC1683130

[B143] Kumar R. Gururaj A. E. Barnes C. J. (2006). p21-activated kinases in cancer. *Nat. Rev. Cancer* 6 459–471. 10.1038/nrc1892 16723992

[B144] Kvajo M. McKellar H. Drew L. J. Lepagnol-Bestel A. M. Xiao L. Levy R. J. (2011). Altered axonal targeting and short-term plasticity in the hippocampus of Disc1 mutant mice. *Proc. Natl. Acad. Sci. U.S.A.* 108 E1349–E1358. 10.1073/pnas.1114113108 22049344 PMC3241761

[B145] Lee F. H. Zai C. C. Cordes S. P. Roder J. C. Wong A. H. (2013). Abnormal interneuron development in disrupted-in-schizophrenia-1 L100P mutant mice. *Mol. Brain* 6:20. 10.1186/1756-6606-6-20 PMC3648430 23631734

[B146] Lee V. M. Goedert M. Trojanowski J. Q. (2001). Neurodegenerative tauopathies. *Annu. Rev. Neurosci.* 24 1121–1159. 10.1146/annurev.neuro.24.1.1121 11520930

[B147] Lev N. Roncevich D. Ickowicz D. Melamed E. Offen D. (2007). Role of DJ-1 in parkinson’s disease. *J. Mol. Neurosci.* 31:307. 10.1385/jmn:31:03:307 17726235

[B148] Levy-Lahad E. Wasco W. Poorkaj P. Romano D. M. Oshima J. Pettingell W. H. (1995). Candidate gene for the chromosome 1 familial Alzheimer’s disease locus. *Science* 269 973–977. 10.1126/science.7638621 7638622

[B149] Lewis J. Dickson D. W. Lin W. L. Chisholm L. Corral A. Jones G. (2001). Enhanced neurofibrillary degeneration in transgenic mice expressing mutant tau and APP. *Science* 293 1487–1491. 10.1126/science.1058189 11520987

[B150] Lewis J. McGowan E. Rockwood J. Melrose H. Nacharaju P. Van Slegtenhorst M. (2000). Neurofibrillary tangles, amyotrophy and progressive motor disturbance in mice expressing mutant (P301L) tau protein. *Nat. Genet.* 25 402–405. 10.1038/78078 10932182

[B151] Li T. Yang D. Sushchky S. Liu Z. Smith W. W. (2011). Models for LRRK2-Linked Parkinsonism. *Parkinsons Dis.* 2011:942412 10.4061/2011/942412 PMC3096154 21603132

[B152] Li X. Patel J. C. Wang J. Avshalumov M. V. Nicholson C. Buxbaum J. D. (2010). Enhanced striatal dopamine transmission and motor performance with LRRK2 overexpression in mice is eliminated by familial Parkinson’s disease mutation G2019S. *J. Neurosci.* 30 1788–1797. 10.1523/JNEUROSCI.5604-09.2010 20130188 PMC2858426

[B153] Li Y. Liu W. Oo T. F. Wang L. Tang Y. Jackson-Lewis V. (2009). Mutant LRRK2(R1441G) BAC transgenic mice recapitulate cardinal features of Parkinson’s disease. *Nat. Neurosci.* 12 826–828. 10.1038/nn.2349 19503083 PMC2845930

[B154] Liang J. Xu W. Hsu Y. T. Yee A. X. Chen L. Sudhof T. C. (2015). Conditional neuroligin-2 knockout in adult medial prefrontal cortex links chronic changes in synaptic inhibition to cognitive impairments. *Mol. Psychiatry* 20 850–859. 10.1038/mp.2015.31 25824299

[B155] Lin X. Parisiadou L. Gu X. L. Wang L. Shim H. Sun L. (2009). Leucine-rich repeat kinase 2 regulates the progression of neuropathology induced by Parkinson’s-disease-related mutant alpha-synuclein. *Neuron* 64 807–827. 10.1016/j.neuron.2009.11.006 20064389 PMC2807409

[B156] Lipina T. V. Wang M. Liu F. Roder J. C. (2012). Synergistic interactions between PDE4B and GSK-3: DISC1 mutant mice. *Neuropharmacology* 62 1252–1262. 10.1016/j.neuropharm.2011.02.020 21376063

[B157] Liu H. F. Lu S. Ho P. W. Tse H. M. Pang S. Y. Kung M. H. (2014). LRRK2 R1441G mice are more liable to dopamine depletion and locomotor inactivity. *Ann. Clin. Transl. Neurol.* 1 199–208. 10.1002/acn3.45 25356398 PMC4184549

[B158] Lok K. Zhao H. Shen H. Wang Z. Gao X. Zhao W. (2013). Characterization of the APP/PS1 mouse model of Alzheimer’s disease in senescence accelerated background. *Neurosci. Lett.* 557(Pt B) 84–89. 10.1016/j.neulet.2013.10.051 24176881

[B159] Lu X. H. Fleming S. M. Meurers B. Ackerson L. C. Mortazavi F. Lo V. (2009). Bacterial artificial chromosome transgenic mice expressing a truncated mutant parkin exhibit age-dependent hypokinetic motor deficits, dopaminergic neuron degeneration, and accumulation of proteinase K-resistant alpha-synuclein. *J. Neurosci.* 29 1962–1976. 10.1523/JNEUROSCI.5351-08.2009 19228951 PMC2803056

[B160] Ma L. Liu Y. Ky B. Shughrue P. J. Austin C. P. Morris J. A. (2002). Cloning and characterization of Disc1, the mouse ortholog of DISC1 (Disrupted-in-Schizophrenia 1). *Genomics* 80 662–672. 10.1006/geno.2002.7012 12504857

[B161] Mandel J. L. Chelly J. (2004). Monogenic X-linked mental retardation: is it as frequent as currently estimated? The paradox of the ARX (Aristaless X) mutations. *Eur. J. Hum. Genet.* 12 689–693. 10.1038/sj.ejhg.5201247 15319782

[B162] Manser E. Leung T. Lim L. (1995). Purification and assay of kinases that interact with Rac/Cdc42. *Methods Enzymol.* 256 215–227. 10.1016/0076-6879(95)56026-2 7476435

[B163] Masliah E. Hansen L. A. (2012). Alzheimer disease: AD pathology–emerging subtypes or age-of-onset spectrum? *Nat. Rev. Neurol.* 8 11–12. 10.1038/nrneurol.2011.205 22143361

[B164] Masliah E. Rockenstein E. Veinbergs I. Mallory M. Hashimoto M. Takeda A. (2000). Dopaminergic loss and inclusion body formation in alpha-synuclein mice: implications for neurodegenerative disorders. *Science* 287 1265–1269. 10.1126/science.287.5456.1265 10678833

[B165] Melrose H. L. Dachsel J. C. Behrouz B. Lincoln S. J. Yue M. Hinkle K. M. (2010). Impaired dopaminergic neurotransmission and microtubule-associated protein tau alterations in human LRRK2 transgenic mice. *Neurobiol. Dis.* 40 503–517. 10.1016/j.nbd.2010.07.010 20659558 PMC2955774

[B166] Meng J. Meng Y. Hanna A. Janus C. Jia Z. (2005). Abnormal long-lasting synaptic plasticity and cognition in mice lacking the mental retardation gene Pak3. *J. Neurosci.* 25 6641–6650. 10.1523/JNEUROSCI.0028-05.2005 16014725 PMC6725420

[B167] Meng Y. Takahashi H. Meng J. Zhang Y. Lu G. Asrar S. (2004). Regulation of ADF/cofilin phosphorylation and synaptic function by LIM-kinase. *Neuropharmacology* 47 746–754. 10.1016/j.neuropharm.2004.06.030 15458846

[B168] Meng Y. Zhang Y. Tregoubov V. Janus C. Cruz L. Jackson M. (2002). Abnormal spine morphology and enhanced LTP in LIMK-1 knockout mice. *Neuron* 35 121–133. 10.1016/S0896-6273(02)00758-4 12123613

[B169] Merla G. Brunetti-Pierri N. Micale L. Fusco C. (2010). Copy number variants at Williams-Beuren syndrome 7q11.*23* region. *Hum. Genet.* 128 3–26. 10.1007/s00439-010-0827-2 20437059

[B170] Mervis C. B. John A. E. (2010). Cognitive and behavioral characteristics of children with Williams syndrome: implications for intervention approaches. *Am. J. Med. Genet. C Semin. Med. Genet.* 154C 229–248. 10.1002/ajmg.c.30263 20425784 PMC2922953

[B171] Mervis C. B. Velleman S. L. (2011). Children with Williams Syndrome: language, cognitive, and behavioral characteristics and their implications for intervention. *Perspect. Lang. Learn. Educ.* 18 98–107. 10.1044/lle18.3.98 22754603 PMC3383614

[B172] Meyer D. Birchmeier C. (1995). Multiple essential functions of neuregulin in development. *Nature* 378 386–390. 10.1038/378386a0 7477375

[B173] Mientjes E. J. Nieuwenhuizen I. Kirkpatrick L. Zu T. Hoogeveen-Westerveld M. Severijnen L. (2006). The generation of a conditional Fmr1 knock out mouse model to study Fmrp function in vivo. *Neurobiol. Dis.* 21 549–555. 10.1016/j.nbd.2005.08.019 16257225

[B174] Millar J. K. Pickard B. S. Mackie S. James R. Christie S. Buchanan S. R. (2005). DISC1 and PDE4B are interacting genetic factors in schizophrenia that regulate cAMP signaling. *Science* 310 1187–1191. 10.1126/science.1112915 16293762

[B175] Missler M. Zhang W. Rohlmann A. Kattenstroth G. Hammer R. E. Gottmann K. (2003). Alpha-neurexins couple Ca2+ channels to synaptic vesicle exocytosis. *Nature* 423 939–948. 10.1038/nature01755 12827191

[B176] Moechars D. Dewachter I. Lorent K. Reverse D. Baekelandt V. Naidu A. (1999). Early phenotypic changes in transgenic mice that overexpress different mutants of amyloid precursor protein in brain. *J. Biol. Chem.* 274 6483–6492. 10.1074/jbc.274.10.6483 10037741

[B177] Mohn A. R. Gainetdinov R. R. Caron M. G. Koller B. H. (1999). Mice with reduced NMDA receptor expression display behaviors related to schizophrenia. *Cell* 98 427–436. 10.1016/S0092-8674(00)81972-8 10481908

[B178] Morris J. A. Kandpal G. Ma L. Austin C. P. (2003). DISC1 (Disrupted-In-Schizophrenia 1) is a centrosome-associated protein that interacts with MAP1A, MIPT3, ATF4/5 and NUDEL: regulation and loss of interaction with mutation. *Hum. Mol. Genet.* 12 1591–1608. 10.1093/hmg/ddg162 12812986

[B179] Morris M. Knudsen G. M. Maeda S. Trinidad J. C. Ioanoviciu A. Burlingame A. L. (2015). Tau post-translational modifications in wild-type and human amyloid precursor protein transgenic mice. *Nat. Neurosci.* 18 1183–1189. 10.1038/nn.4067 26192747 PMC8049446

[B180] Naisbitt S. Kim E. Tu J. C. Xiao B. Sala C. Valtschanoff J. (1999). Shank, a novel family of postsynaptic density proteins that binds to the NMDA receptor/PSD-95/GKAP complex and cortactin. *Neuron* 23 569–582. 10.1016/S0896-6273(00)80809-0 10433268

[B181] Naslund J. Haroutunian V. Mohs R. Davis K. L. Davies P. Greengard P. (2000). Correlation between elevated levels of amyloid beta-peptide in the brain and cognitive decline. *JAMA* 283 1571–1577. 10.1001/jama.283.12.1571 10735393

[B182] Nichols W. C. Pankratz N. Hernandez D. Paisan-Ruiz C. Jain S. Halter C. A. (2005). Genetic screening for a single common LRRK2 mutation in familial Parkinson’s disease. *Lancet* 365 410–412. 10.1016/S0140-6736(05)17828-3 15680455

[B183] No authors (1994). Fmr1 knockout mice: a model to study fragile X mental retardation. The Dutch-Belgian Fragile X Consortium. *Cell* 78 23–33. 8033209

[B184] O’Brien R. J. Wong P. C. (2011). Amyloid precursor protein processing and Alzheimer’s disease. *Annu. Rev. Neurosci.* 34 185–204. 10.1146/annurev-neuro-061010-113613 21456963 PMC3174086

[B185] Oddo S. Caccamo A. Kitazawa M. Tseng B. P. LaFerla F. M. (2003). Amyloid deposition precedes tangle formation in a triple transgenic model of Alzheimer’s disease. *Neurobiol. Aging* 24 1063–1070. 10.1016/j.neurobiolaging.2003.08.012 14643377

[B186] O’Donnell W. T. Warren S. T. (2002). A decade of molecular studies of fragile X syndrome. *Annu. Rev. Neurosci.* 25 315–338. 10.1146/annurev.neuro.25.112701.142909 12052912

[B187] O’Tuathaigh C. M. Babovic D. O’Meara G. Clifford J. J. Croke D. T. Waddington J. L. (2007). Susceptibility genes for schizophrenia: characterisation of mutant mouse models at the level of phenotypic behaviour. *Neurosci. Biobehav. Rev.* 31 60–78. 10.1016/j.neubiorev.2006.04.002 16782199

[B188] Pankratz N. Nichols W. C. Elsaesser V. E. Pauciulo M. W. Marek D. K. Halter C. A. (2009). Alpha-synuclein and familial Parkinson’s disease. *Mov. Disord.* 24 1125–1131. 10.1002/mds.22524 19412953 PMC3397145

[B189] Parisiadou L. Xie C. Cho H. J. Lin X. Gu X. L. Long C. X. (2009). Phosphorylation of ezrin/radixin/moesin proteins by LRRK2 promotes the rearrangement of actin cytoskeleton in neuronal morphogenesis. *J. Neurosci.* 29 13971–13980. 10.1523/JNEUROSCI.3799-09.2009 19890007 PMC2807632

[B190] Parisiadou L. Yu J. Sgobio C. Xie C. Liu G. Sun L. (2014). LRRK2 regulates synaptogenesis and dopamine receptor activation through modulation of PKA activity. *Nat. Neurosci.* 17 367–376. 10.1038/nn.3636 24464040 PMC3989289

[B191] Peca J. Feliciano C. Ting J. T. Wang W. Wells M. F. Venkatraman T. N. (2011). Shank3 mutant mice display autistic-like behaviours and striatal dysfunction. *Nature* 472 437–442. 10.1038/nature09965 21423165 PMC3090611

[B192] Penagarikano O. Abrahams B. S. Herman E. I. Winden K. D. Gdalyahu A. Dong H. (2011). Absence of CNTNAP2 leads to epilepsy, neuronal migration abnormalities, and core autism-related deficits. *Cell* 147 235–246. 10.1016/j.cell.2011.08.040 21962519 PMC3390029

[B193] Perez F. A. Palmiter R. D. (2005). Parkin-deficient mice are not a robust model of parkinsonism. *Proc. Natl. Acad. Sci. U.S.A.* 102 2174–2179. 10.1073/pnas.0409598102 15684050 PMC548311

[B194] Petersen R. C. Smith G. E. Waring S. C. Ivnik R. J. Tangalos E. G. Kokmen E. (1999). Mild cognitive impairment: clinical characterization and outcome. *Arch. Neurol.* 56 303–308. 10.1001/archneur.56.3.303 10190820

[B195] Phelan M. C. Rogers R. C. Saul R. A. Stapleton G. A. Sweet K. McDermid H. (2001). 22q13 deletion syndrome. *Am. J. Med. Genet.* 101 91–99. 10.1002/1096-8628(20010615)101:2<91::AID-AJMG1340>3.0.CO;2-C 11391650

[B196] Pietropaolo S. Subashi E. (2014). “Fragile X syndrome,” in *Behavioral Genetics of the Mouse: Genetic Mouse Models of Neurobehavioural Disorders* eds Pietropaolo F. S. S. Crusio W. E. (Cambridge: Cambridge University Press).

[B197] Plaas M. Karis A. Innos J. Rebane E. Baekelandt V. Vaarmann A. (2008). Alpha-synuclein A30P point-mutation generates age-dependent nigrostriatal deficiency in mice. *J. Physiol. Pharmacol.* 59 205–216. 18622040

[B198] Pober B. R. (2010). Williams-Beuren syndrome. *N. Engl. J. Med.* 362 239–252. 10.1056/NEJMra0903074 20089974

[B199] Poliak S. Gollan L. Martinez R. Custer A. Einheber S. Salzer J. L. (1999). Caspr2, a new member of the neurexin superfamily, is localized at the juxtaparanodes of myelinated axons and associates with K+ channels. *Neuron* 24 1037–1047. 10.1016/S0896-6273(00)81049-1 10624965

[B200] Polymeropoulos M. H. Lavedan C. Leroy E. Ide S. E. Dehejia A. Dutra A. (1997). Mutation in the alpha-synuclein gene identified in families with Parkinson’s disease. *Science* 276 2045–2047. 10.1126/science.276.5321.2045 9197268

[B201] Rabaneda L. G. Robles-Lanuza E. Nieto-Gonzalez J. L. Scholl F. G. (2014). Neurexin dysfunction in adult neurons results in autistic-like behavior in mice. *Cell Rep.* 8 338–346. 10.1016/j.celrep.2014.06.022 25017069

[B202] Radyushkin K. Hammerschmidt K. Boretius S. Varoqueaux F. El-Kordi A. Ronnenberg A. (2009). Neuroligin-3-deficient mice: model of a monogenic heritable form of autism with an olfactory deficit. *Genes Brain Behav.* 8 416–425. 10.1111/j.1601-183X.2009.00487.x 19243448

[B203] Reyniers L. Del Giudice M. G. Civiero L. Belluzzi E. Lobbestael E. Beilina A. (2014). Differential protein-protein interactions of LRRK1 and LRRK2 indicate roles in distinct cellular signaling pathways. *J. Neurochem.* 131 239–250. 10.1111/jnc.12798 24947832 PMC4272680

[B204] Rial D. Castro A. A. Machado N. Garcao P. Goncalves F. Q. Silva H. B. (2014). Behavioral phenotyping of Parkin-deficient mice: looking for early preclinical features of Parkinson’s disease. *PLoS ONE* 9:e114216 10.1371/journal.pone.0114216 PMC4259468 25486126

[B205] Rieker C. Dev K. K. Lehnhoff K. Barbieri S. Ksiazek I. Kauffmann S. (2011). Neuropathology in mice expressing mouse alpha-synuclein. *PLoS ONE* 6:e24834 10.1371/journal.pone.0024834 PMC3180287 21966373

[B206] Rockenstein E. Mallory M. Hashimoto M. Song D. Shults C. W. Lang I. (2002). Differential neuropathological alterations in transgenic mice expressing alpha-synuclein from the platelet-derived growth factor and Thy-1 promoters. *J. Neurosci. Res.* 68 568–578. 10.1002/jnr.10231 12111846

[B207] Rodenas-Cuadrado P. Ho J. Vernes S. C. (2014). Shining a light on CNTNAP2: complex functions to complex disorders. *Eur. J. Hum. Genet.* 22 171–178. 10.1038/ejhg.2013.100 23714751 PMC3895625

[B208] Roder S. Danober L. Pozza M. F. Lingenhoehl K. Wiederhold K. H. Olpe H. R. (2003). Electrophysiological studies on the hippocampus and prefrontal cortex assessing the effects of amyloidosis in amyloid precursor protein 23 transgenic mice. *Neuroscience* 120 705–720. 10.1016/S0306-4522(03)00381-6 12895511

[B209] Rothwell P. E. Fuccillo M. V. Maxeiner S. Hayton S. J. Gokce O. Lim B. K. (2014). Autism-associated neuroligin-3 mutations commonly impair striatal circuits to boost repetitive behaviors. *Cell* 158 198–212. 10.1016/j.cell.2014.04.045 24995986 PMC4120877

[B210] Sachs N. A. Sawa A. Holmes S. E. Ross C. A. DeLisi L. E. Margolis R. L. (2005). A frameshift mutation in Disrupted in Schizophrenia 1 in an American family with schizophrenia and schizoaffective disorder. *Mol. Psychiatry* 10 758–764. 10.1038/sj.mp.4001667 15940305

[B211] Sanders S. J. Ercan-Sencicek A. G. Hus V. Luo R. Murtha M. T. Moreno-De-Luca D. (2011). Multiple recurrent de novo CNVs, including duplications of the 7q11.*23* Williams syndrome region, are strongly associated with autism. *Neuron* 70 863–885. 10.1016/j.neuron.2011.05.002 21658581 PMC3939065

[B212] Satake W. Nakabayashi Y. Mizuta I. Hirota Y. Ito C. Kubo M. (2009). Genome-wide association study identifies common variants at four loci as genetic risk factors for Parkinson’s disease. *Nat. Genet.* 41 1303–1307. 10.1038/ng.485 19915576

[B213] Saura C. A. Chen G. Malkani S. Choi S. Y. Takashashi R. H. Zhang D. (2005). Conditional inactivation of presenilin 1 prevents amyloid accumulation and temporarily rescues contextual and spatial working memroy impairments in amyloid precursor protein transgenic mice. *J. Neurosci.* 29 6755–6764. 10.1523/JNEUROSCI.1247-05.2005 16033885 PMC6725351

[B214] Savonenko A. V. Xu G. M. Price D. L. Borchelt D. R. Markowska A. L. (2003). Normal cognitive behavior in two distinct congenic lines of transgenic mice hyperexpressing mutant APP SWE. *Neurobiol. Dis.* 12 194–211. 10.1016/S0969-9961(02)00012-8 12742740

[B215] Scheuner D. Eckman C. Jensen M. Song X. Citron M. Suzuki N. (1996). Secreted amyloid beta-protein similar to that in the senile plaques of Alzheimer’s disease is increased in vivo by the presenilin 1 and 2 and APP mutations linked to familial Alzheimer’s disease. *Nat. Med.* 2 864–870. 10.1038/nm0896-864 8705854

[B216] Schughart K. Libert C. Kas M. J. (2013). Controlling complexity: the clinical relevance of mouse complex genetics. *Eur. J. Hum. Genet.* 21 1191–1196. 10.1038/ejhg.2013.79 23632795 PMC3798854

[B217] Sherrington R. Rogaev E. I. Liang Y. Rogaeva E. A. Levesque G. Ikeda M. (1995). Cloning of a gene bearing missense mutations in early-onset familial Alzheimer’s disease. *Nature* 375 754–760. 10.1038/375754a0 7596406

[B218] Simon A. M. Schiapparelli L. Salazar-Colocho P. Cuadrado-Tejedor M. Escribano L. Lopez de Maturana R. (2009). Overexpression of wild-type human APP in mice causes cognitive deficits and pathological features unrelated to Abeta levels. *Neurobiol. Dis.* 33 369–378. 10.1016/j.nbd.2008.11.005 19101630

[B219] Singleton A. B. (2005). Altered alpha-synuclein homeostasis causing Parkinson’s disease: the potential roles of dardarin. *Trends Neurosci.* 28 416–421. 10.1016/j.tins.2005.05.009 15955578

[B220] Soares D. C. Carlyle B. C. Bradshaw N. J. Porteous D. J. (2011). DISC1: structure, function, and therapeutic potential for major mental illness. *ACS Chem. Neurosci.* 2 609–632. 10.1021/cn200062k 22116789 PMC3222219

[B221] Sorensen E. M. Bertelsen F. Weikop P. Skovborg M. M. Banke T. Drasbek K. R. (2015). Hyperactivity and lack of social discrimination in the adolescent Fmr1 knockout mouse. *Behav. Pharmacol.* 26 733–740. 10.1097/FBP.0000000000000152 26110222

[B222] Speed H. E. Kouser M. Xuan Z. Reimers J. M. Ochoa C. F. Gupta N. (2015). Autism-associated insertion mutation (InsG) of Shank3 Exon 21 causes impaired synaptic transmission and behavioral deficits. *J. Neurosci.* 35 9648–9665. 10.1523/JNEUROSCI.3125-14.2015 26134648 PMC4571502

[B223] Spencer C. M. Serysheva E. Yuva-Paylor L. A. Oostra B. A. Nelson D. L. Paylor R. (2006). Exaggerated behavioral phenotypes in Fmr1/Fxr2 double knockout mice reveal a functional genetic interaction between Fragile X-related proteins. *Hum. Mol. Genet.* 15 1984–1994. 10.1093/hmg/ddl121 16675531

[B224] Sperling R. A. Aisen P. S. Beckett L. A. Bennett D. A. Craft S. Fagan A. M. (2011). Toward defining the preclinical stages of Alzheimer’s disease: recommendations from the National Institute on Aging-Alzheimer’s Association workgroups on diagnostic guidelines for Alzheimer’s disease. *Alzheimers Dement.* 7 280–292. 10.1016/j.jalz.2011.03.003 21514248 PMC3220946

[B225] Spillantini M. G. Crowther R. A. Jakes R. Cairns N. J. Lantos P. L. Goedert M. (1998). Filamentous alpha-synuclein inclusions link multiple system atrophy with Parkinson’s disease and dementia with Lewy bodies. *Neurosci. Lett.* 251 205–208. 10.1016/S0304-3940(98)00504-7 9726379

[B226] St Clair D. Blackwood D. Muir W. Carothers A. Walker M. Spowart G. (1990). Association within a family of a balanced autosomal translocation with major mental illness. *Lancet* 336 13–16. 10.1016/0140-6736(90)91520-K 1973210

[B227] Stefansson H. Sigurdsson E. Steinthorsdottir V. Bjornsdottir S. Sigmundsson T. Ghosh S. (2002). Neuregulin 1 and susceptibility to schizophrenia. *Am. J. Hum. Genet.* 71 877–892. 10.1086/342734 12145742 PMC378543

[B228] Stromme P. Bjornstad P. G. Ramstad K. (2002). Prevalence estimation of Williams syndrome. *J. Child Neurol.* 17 269–271. 10.1177/088307380201700406 12088082

[B229] Sturchler-Pierrat C. Abramowski D. Duke M. Wiederhold K. H. Mistl C. Rothacher S. (1997). Two amyloid precursor protein transgenic mouse models with Alzheimer disease-like pathology. *Proc. Natl. Acad. Sci. U.S.A.* 94 13287–13292. 10.1073/pnas.94.24.13287 9371838 PMC24301

[B230] Sudhof T. C. (2008). Neuroligins and neurexins link synaptic function to cognitive disease. *Nature* 455 903–911. 10.1038/nature07456 18923512 PMC2673233

[B231] Sumi T. Matsumoto K. Takai Y. Nakamura T. (1999). Cofilin phosphorylation and actin cytoskeletal dynamics regulated by rho- and Cdc42-activated LIM-kinase 2. *J. Cell Biol.* 147 1519–1532. 10.1083/jcb.147.7.1519 10613909 PMC2174243

[B232] Tabuchi K. Blundell J. Etherton M. R. Hammer R. E. Liu X. Powell C. M. (2007). A neuroligin-3 mutation implicated in autism increases inhibitory synaptic transmission in mice. *Science* 318 71–76. 10.1126/science.1146221 17823315 PMC3235367

[B233] Tabuchi K. Sudhof T. C. (2002). Structure and evolution of neurexin genes: insight into the mechanism of alternative splicing. *Genomics* 79 849–859. 10.1006/geno.2002.6780 12036300

[B234] Tassabehji M. Metcalfe K. Fergusson W. D. Carette M. J. Dore J. K. Donnai D. (1996). LIM-kinase deleted in Williams syndrome. *Nat. Genet.* 13 272–273. 10.1038/ng0796-272 8673124

[B235] Thiruchelvam M. J. Powers J. M. Cory-Slechta D. A. Richfield E. K. (2004). Risk factors for dopaminergic neuron loss in human alpha-synuclein transgenic mice. *Eur. J. Neurosci.* 19 845–854. 10.1111/j.0953-816X.2004.03139.x 15009131

[B236] Todorovski Z. Asrar S. Liu J. Saw N. M. Joshi K. Cortez M. A. (2015). LIMK1 regulates long-term memory and synaptic plasticity via the transcriptional factor CREB. *Mol. Cell. Biol.* 35 1316–1328. 10.1128/MCB.01263-14 25645926 PMC4372699

[B237] Tong Y. Shen J. (2009). alpha-synuclein and LRRK2: partners in crime. *Neuron* 64 771–773. 10.1016/j.neuron.2009.12.017 20064381 PMC2826885

[B238] Uchino S. Waga C. (2013). SHANK3 as an autism spectrum disorder-associated gene. *Brain Dev.* 35 106–110. 10.1016/j.braindev.2012.05.013 22749736

[B239] Um J. W. Stichel-Gunkel C. Lubbert H. Lee G. Chung K. C. (2009). Molecular interaction between parkin and PINK1 in mammalian neuronal cells. *Mol. Cell. Neurosci.* 40 421–432. 10.1016/j.mcn.2008.12.010 19167501

[B240] van der Putten H. Wiederhold K. H. Probst A. Barbieri S. Mistl C. Danner S. (2000). Neuropathology in mice expressing human alpha-synuclein. *J. Neurosci.* 20 6021–6029. 10934251 10.1523/JNEUROSCI.20-16-06021.2000 PMC6772584

[B241] Varoqueaux F. Aramuni G. Rawson R. L. Mohrmann R. Missler M. Gottmann K. (2006). Neuroligins determine synapse maturation and function. *Neuron* 51 741–754. 10.1016/j.neuron.2006.09.003 16982420

[B242] Verkerk A. J. Pieretti M. Sutcliffe J. S. Fu Y. H. Kuhl D. P. Pizzuti A. (1991). Identification of a gene (FMR-1) containing a CGG repeat coincident with a breakpoint cluster region exhibiting length variation in fragile X syndrome. *Cell* 65 905–914. 10.1016/0092-8674(91)90397-H 1710175

[B243] Vigo-Pelfrey C. Lee D. Keim P. Lieberburg I. Schenk D. B. (1993). Characterization of beta-amyloid peptide from human cerebrospinal fluid. *J. Neurochem.* 61 1965–1968. 10.1111/j.1471-4159.1993.tb09841.x 8229004

[B244] West A. B. Moore D. J. Biskup S. Bugayenko A. Smith W. W. Ross C. A. (2005). Parkinson’s disease-associated mutations in leucine-rich repeat kinase 2 augment kinase activity. *Proc. Natl. Acad. Sci. U.S.A.* 102 16842–16847. 10.1073/pnas.0507360102 16269541 PMC1283829

[B245] Winner B. Melrose H. L. Zhao C. Hinkle K. M. Yue M. Kent C. (2011). Adult neurogenesis and neurite outgrowth are impaired in LRRK2 G2019S mice. *Neurobiol. Dis.* 41 706–716. 10.1016/j.nbd.2010.12.008 21168496 PMC3059106

[B246] Yang N. Higuchi O. Ohashi K. Nagata K. Wada A. Kangawa K. (1998). Cofilin phosphorylation by LIM-kinase 1 and its role in Rac-mediated actin reorganization. *Nature* 393 809–812. 10.1038/31735 9655398

[B247] Yu H. Saura C. A. Choi S. Y. Sun L. D. Yang X. Handler M. (2001). APP processing and synaptic plasticity in presenilin-1 conditional knock-out mice. *Neuron* 31 713–726. 10.1016/S0896-6273(01)00417-2 11567612

[B248] Yuhas J. Cordeiro L. Tassone F. Ballinger E. Schneider A. Long J. M. (2011). Brief report: sensorimotor gating in idiopathic autism and autism associated with fragile X syndrome. *J. Autism. Dev. Disord.* 41 248–253. 10.1007/s10803-010-1040-9 20521090 PMC3023021

[B249] Zhang B. R. Hu Z. X. Yin X. Z. Cai M. Zhao G. H. Liu Z. R. (2010). Mutation analysis of parkin and PINK1 genes in early-onset Parkinson’s disease in China. *Neurosci. Lett.* 477 19–22. 10.1016/j.neulet.2010.04.026 20399249

[B250] Zhao L. Ma Q. L. Calon F. Harris-White M. E. Yang F. Lim G. P. (2006). Role of p21-activated kinase pathway defects in the cognitive deficits of Alzheimer disease. *Nat. Neurosci.* 9 234–242. 10.1038/nn1630 16415866

[B251] Zhou H. Falkenburger B. H. Schulz J. B. Tieu K. Xu Z. Xia X. G. (2007). Silencing of the Pink1 gene expression by conditional RNAi does not induce dopaminergic neuron death in mice. *Int. J. Biol. Sci.* 3 242–250. 10.7150/ijbs.3.242 17389931 PMC1820878

